# Resonant acoustic mixing-assisted fabrication and evaluation of FDM-printed PLA/β-TCP scaffolds for bone tissue engineering

**DOI:** 10.3389/fbioe.2025.1691481

**Published:** 2025-11-25

**Authors:** Yixuan Zhu, Jiangqi Hu, Bin Luo, Xuening Deng, Siyu Xie, Jiuning Huang, Yafei Yuan, Qingsong Jiang

**Affiliations:** 1 School of Stomatology, Capital Medical University, Beijing, China; 2 Department of Prosthodontics, Beijing Stomatological Hospital, Capital Medical University, Beijing, China; 3 Faculty of Materials Science and Engineering, Beijing University of Technology, Beijing, China

**Keywords:** bone tissue engineering, 3D printing, polylactic acid, β-tricalcium phosphate, resonant acoustic mixing, composite scaffolds

## Abstract

**Background:**

Composite scaffolds combining polylactic acid (PLA) with β-tricalcium phosphate (β-TCP) offer potential for bone tissue engineering (BTE) by integrating mechanical support with bioactivity. However, the optimal ratio balancing biological performance, structural integrity, and manufacturing feasibility remains unclear.

**Methods:**

PLA/β-TCP composites containing 0%, 10%, 20%, and 30% β-TCP were prepared using solvent-free resonant acoustic mixing (RAM), extruded into filaments, and printed by fused deposition modeling (FDM). Scaffolds were evaluated for mechanical properties, printability, and *in vitro* biocompatibility with rat bone marrow mesenchymal stem cells (rBMSCs). Osteogenic differentiation was assessed by ALP activity, calcium deposition, and expression of osteogenic marker. *In vivo* bone regeneration was investigated in a rat calvarial defect model.

**Results:**

All β-TCP-containing scaffolds enhanced cell adhesion, proliferation, and osteogenesis compared with pure PLA. The 80:20 PLA/β-TCP scaffold showed optimal balance of bioactivity, compressive strength, and printing quality. Excessive β-TCP (>20%) reduced mechanical strength and caused printing defects. *In vivo*, the 80:20 group showed superior early bone regeneration after 4 weeks, confirmed by micro-CT and histology.

**Conclusion:**

The 80:20 PLA/β-TCP composition offers an optimal balance of biological activity, mechanical performance, and manufacturing scalability, supporting its potential as a cost-effective scaffold for clinical BTE applications.

## 1 Introduction

Although bone tissue has a strong self-healing capacity, critical-size bone defects caused by tumors, trauma, infections, etc., cannot heal spontaneously. Their reconstruction and repair still require bone grafting techniques, which remains an unmet clinical need (Huang et al., 2022). Main bone grafting techniques include autologous bone grafting, allogeneic bone grafting, xenogeneic bone grafting, and artificial bone grafting. The autologous bone grafting, due to its high immunocompatibility, is considered the gold standard for most applications in orthopedics (Zhang et al., 2024). The drawbacks of autologous bone grafting include establishment of a second surgical area, limited bone supply, and complications associated with pain, nerve injury, hematoma formation, and infection, etc. (Simonds et al., 1992). Bone tissue engineering (BTE) aims to achieve the repair and regeneration of functional bone through the synergistic effect of biomaterials, cells, and signaling molecules. Biomaterials play a crucial role in BTE by providing a porous scaffold that supports early mechanical loading and guides the continuous growth of the defected bones while gradually degrading over time. Ultimately, this process results in the permanent repair and regeneration of functional bone within the scaffold region (Amini et al., 2012).

Among various bone scaffolds fabrication methods, traditional techniques such as solvent casting and particulate leaching often struggle to conform to the morphology of bone defects and lack controllable porosity. Techniques involving hydrogel or bone cement injection can conform well to bone defect sites but suffer from limitations such as insufficient mechanical strength and difficulty in fixation (Zhang et al., 2018). 3D-printed bone scaffolds offer a novel solution to the challenges of bone grafting, with unparalleled advantages over traditional artificial bone scaffolds in terms of controllability of pore size, porosity, and mechanical properties. And computer-assisted surgical simulation, coupled with advancements in 3D printed BTE scaffolds, enables surgeons to virtually plan cases, create personalized surgical models, design 3D-printed bone scaffolds tailored to the dimensions of mandibular defects (Bouyer et al., 2021).

In the selection of materials for 3D-printed bone scaffolds, thermoplastic polymers have attracted widespread attention due to their excellent printability and molding stability. Among them, polylactic acid (PLA), a synthetic polymer with excellent biocompatibility and processibility, has been approved by Food and Drug Administration (FDA) for direct contacting with biological fluids (Rasal et al., 2010). PLA-based suture devices, resorbable fracture fixation plates and scaffolds for ligament, cartilage, and bone regeneration suture devices have been widely used in the field of biomedicine (DeStefano et al., 2020). However, previous studies have shown that PLA, when used alone, exhibits strong hydrophobicity and poor affinity with cells and drugs (Ratner, 1995). And aseptic inflammatory complications can occur if the surrounding tissue cannot eliminate the acid by-products during degradation (Stankevich et al., 2015), which may limit the osteoconductive and osteoinductive capabilities of PLA implants. Therefore, PLA modification, which primarily includes physical modification and chemical modification, is crucial to increase its applicability (Wu et al., 2020). Although physical modification such as blending relies on weaker interactions such as hydrogen bonding and van der Waals forces rather than strong chemical bonds, it offers a relatively simpler process compared to chemical modification (Chen H. et al., 2018). Additionally, physical modification allows to tailor the biological and mechanical properties of PLA-based composites (Donate et al., 2020).

β-Tricalcium phosphate (β-TCP) is a type of bioceramic with a chemical composition similar to hydroxyapatite (HA) in bone tissue. Its solubility closely matches that of bone minerals, making it resorbable and easily replaceable by new bone (Bohner et al., 2020). Due to its biocompatibility during degradation and dissolution, coupled with a strong affinity for progenitor cells of osteoblasts (Mohammed et al., 2023), β-TCP has emerged as one of the most promising bone graft substitute materials. However, the brittleness exhibited by β-TCP when used alone, like other ceramic materials, raises concerns about its mechanical stability (Ryan and Yin, 2022). Therefore, although the incorporation of β-TCP into the PLA matrix for 3D-printed bone tissue scaffolds has been widely investigated, most existing studies focus on limited property evaluations or single testing modalities. To date, no comprehensive work has systematically examined the *in-vitro* and *in-vivo* performance of scaffolds across different β-TCP loadings, particularly with respect to in-depth mechanical behavior analysis and its correlation with microstructural features. Furthermore, the influence of composition ratio on processing feasibility and production scalability remains insufficiently explored, leaving a gap in translating these composites into clinically applicable manufacturing processes (Backes et al., 2021; Nayak et al., 2024; Hee-Jung et al., 2021; Wang et al., 2024; Salamanca et al., 2021).

At present, the primary 3D printing technique used for fabricating PLA-based bone tissue scaffolds is fused deposition modeling (FDM). FDM printers lead plastic filament to an extruder head where it is melted and forced out through a small diameter jet onto the surface, where it solidifies (Ishida et al., 2020). The excellent affordability, customization and material diversity of FDM make it an attracting technique to fast-fabricate complex and functional engineered bone scaffolds to repair the damaged bone tissues (Raj et al., 2024). However, the uniform dispersion of ceramic particles during filament fabrication represents an additional processing challenge, as density mismatch and agglomeration tend to compromise mechanical performance and printability. Thus, adopting a robust premixing strategy is critical for ensuring reproducibility and scalability. Resonant Acoustic Mixing (RAM) is a new mixing technology based on the coupling effects of macroscopic mixing through vibration and microscopic mixing through acoustic field (Hope et al., 2015). The RAM can transfer mechanical energy created in the springs to the loose mass in the vessel by the propagation of an acoustic pressure wave and was proved as a fine choice to produce powder blends with good content uniformity (Osorio et al., 2016). It can also avoid the use of solvents like acetone, dichloromethane and chloroform, thereby preventing potential cytotoxic effects caused by residual solvents in the system.

**FIGURE 1:**
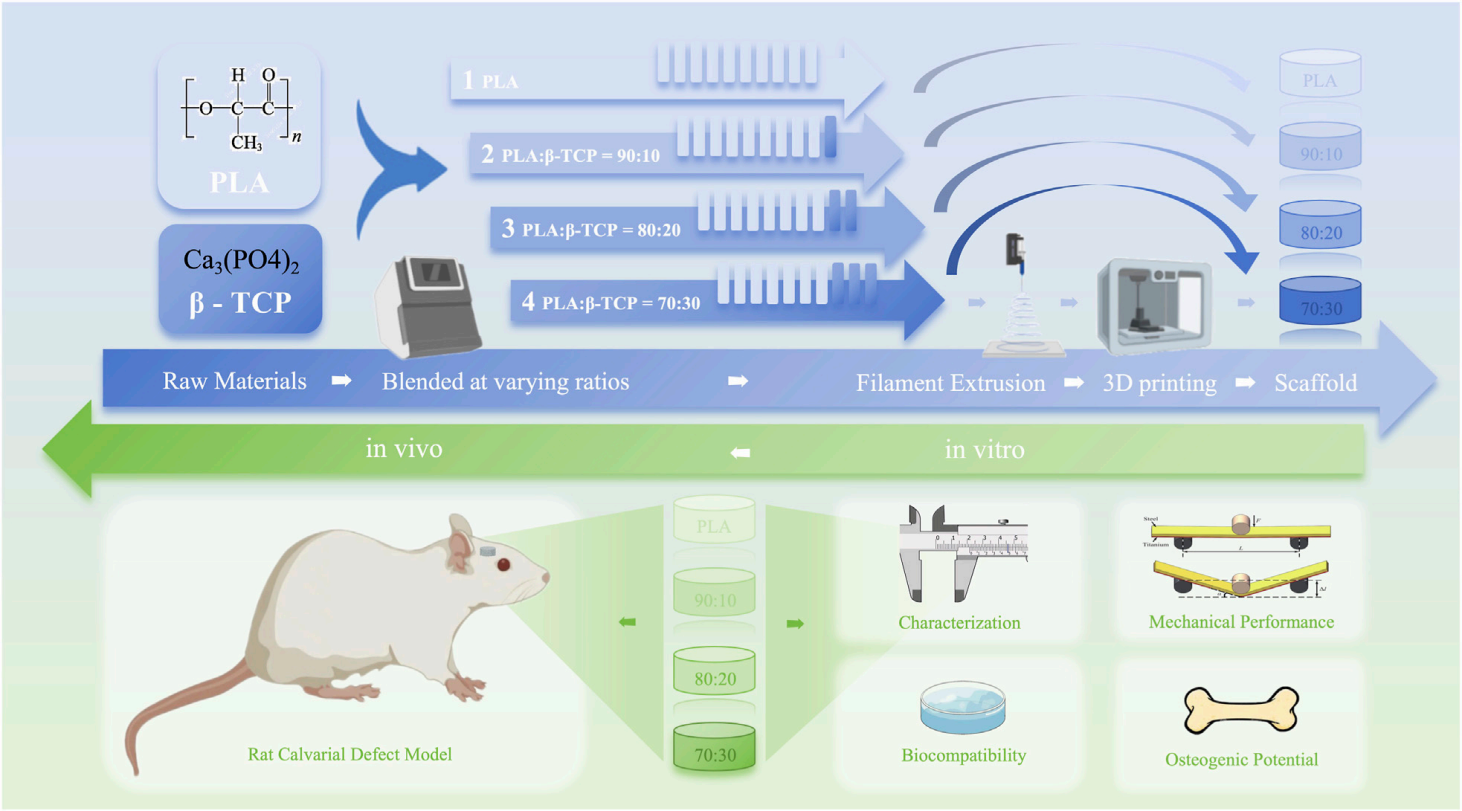
Schematic diagram of the research idea.

In this study, as shown in Figure 1, 3D-printed PLA/β-TCP scaffolds with different β-TCP proportions (0, 10, 20, and 30 wt%) were prepared using FDM and characterized, and the osteogenic property was evaluated both *in vitro* and *in vivo*, providing a reference for the application of these two materials in the field of 3D-printed BTE scaffolds.

## 2 Materials and methods

### 2.1 Materials

Polylactic acid (PLA) powder (product code: 4032D; NatureWorks LLC, USA) was purchased for use. β-tricalcium phosphate (β-TCP) powder (CAS No. 7758-87-4) was obtained from Kunshan Overseas Chinese Science and Technology New Materials Co., Ltd. (China). Transwell inserts were supplied by BIOFIL (China). Penicillin-streptomycin solution, phosphate-buffered saline (PBS), simulated body fluid (SBF), β-glycerophosphate disodium salt, vitamin C, dexamethasone, and glutaraldehyde fixative for electron microscopy were purchased from Solarbio (China). α-Minimum Essential Medium (α-MEM), fetal bovine serum (FBS), and trypsin were sourced from Gibco (USA). Cell Counting Kit-8 (CCK-8), AM/PI live/dead staining kit, Actin-Tracker Red-555, antifade mounting medium with DAPI, BCA protein assay kit, TRIzol reagent and RIPA lysis buffer were obtained from Beyotime Biotechnology (China). SYBR Green Master Mix was purchased from Yeason (China). Primary antibodies against BMP-2, OCN, ALP, and Runx2 were purchased from Abcam (UK).

Rat bone marrow-derived mesenchymal stem cells (rBMSCs) were used for *in vitro* experiments and obtained from ATCC (USA). Sprague-Dawley (SD) rats (male, 12 weeks old) used for *in vivo* experiments were provided by Hangzhou Qizhen Laboratory Animal Sales Co., Ltd. (China), with animal qualification certificate No. [SYXK (ZHE) 2023-0002]. Animal use was approved by the Ethics Committee of Capital Medical University.

### 2.2 Preparation of PLA/β-TCP composite powder

β-TCP powder was mixed into PLA powder at 10wt%, 20 wt%, and 30 wt%, respectively, using a LabSEM-1 high-efficiency resonant mixer (Hangpeng Chemical Power Technology, Hubei, China). The mixing process was conducted at an acceleration of 60G, a vibration frequency of 63 Hz, and a vibration intensity of 50% for 10 min to obtain PLA/β-TCP composite powders, with pure PLA powder used as a control.

### 2.3 Preparation of PLA and PLA/β-TCP filaments

The pure PLA powder and composite powders with different β-TCP ratios were vacuum-dried in an oven at 50 °C for 24 h. The powders were melted and extruded in a single-screw extruder (assembled by Beijing University of Technology) as a 1.75-mm diameter filamentous material, which were then dried and stored for later use. The hopper temperature, extrusion temperature and filament diameter of the extruder were set at 185 °C, 180 °C, and 1.75 ± 0.05 mm.

### 2.4 Preparation of PLA and PLA/β-TCP scaffolds

Printing was performed on a desktop FDM printer (Bambu Lab A1 mini, Shenzhen, China) with filaments previously prepared as raw material using the following printing parameters: nozzle size, 0.4 mm; layer height, 0.2 mm; speed, 10 mm/s. During the printing process of each scaffold group, the heated bed temperature was uniformly set to 60 °C. The nozzle temperatures were set as follows: 210 °C for the PLA group, 205 °C for the 90:10 group, 200 °C for the 80:20 group, and 190 °C for the 70:30 group. The nozzle temperatures for each group were determined based on the optimal print quality achieved through trial and error.

### 2.5 Characterization methods

#### 2.5.1 Particle size analysis of PLA and β -TCP powder

Particle size distribution of PLA and β -TCP (Huaqiao technology New Material, Kunshan, China) powder was measured on Mastersizer 2000 Laser Particle Analyzer (MALVERN, UK) using photon correlation spectroscopy (PSC). Data was analysed and plotted using Origin 2024.

#### 2.5.2 Phase and functional group analysis of the materials

An X-ray diffractometer (XRD) analyzer (Rigaku SmartLab SE, Japan) was used for phase analysis. Functional groups of the materials were characterized by Fourier-transform infrared spectroscopy (FTIR; Thermo Scientific Nicolet 6700, USA) with attenuated total reflectance (ATR).

#### 2.5.3 Surface morphology of the filaments and scaffolds

A layer of gold film of 15 nm thickness was sputtered on the surfaces of PLA and PLA/β-TCP filaments and scaffolds. Macroscopic images of the filaments and scaffolds were captured using a mirrorless camera (Sony A7C2, Japan). For microscopic observation, scanning electron microscopy (SEM; ZEISS Sigma 360, Germany) was performed at an accelerating voltage of 3 kV under high vacuum.

To further evaluate the dispersion of β-TCP particles within the PLA matrix, cross-sectional SEM imaging was conducted using the freeze-fracture method. Filaments from each group were immersed in liquid nitrogen for approximately 5 min and then fractured sharply to expose the internal microstructure without introducing mechanical deformation. The fractured surfaces were sputter-coated with a ∼10 nm gold layer and examined using a scanning electron microscope (TESCAN MIRA LMS, Czech Republic) to characterize internal particle distribution and interfacial bonding. Energy-dispersive X-ray spectroscopy (EDS) was performed on the same SEM system. Point analysis was first conducted on selected regions of interest to verify the local elemental composition. Subsequently, elemental mapping of carbon (C), oxygen (O), calcium (Ca), and phosphorus (P) was carried out on representative cross sections to visualize the spatial distribution of inorganic particles and provide complementary evidence of dispersion uniformity across composite groups.

#### 2.5.4 Hydrophilicity test of the materials

The water contact angle (WCA) of the 3D-printed discs (25 mm × 1 mm, diameter × height) was measured using a contact angle goniometer (Chengde DingSheng JY-82C). Each sample was tested three times at different locations on its surface. Photographs were taken after water droplets were applied to the sample surface, and the contact angle was measured and then calculated using Prism 10.0.

#### 2.5.5 Mechanical tests

Compression tests were performed following the ASTM D695-10 standard and tensile tests were carried out following the ASTM D638-14 standard on the universal testing machine (INSTRON RGM-6300, America) at a velocity of 1.0 mm/min. Each mechanical test (tensile and compression) was performed with three replicates. Photographs of the standard specimens were taken during mechanical testing.

In addition to the tests performed under ambient (dry) conditions, supplementary compression tests with three replicates were carried out under physiological conditions to evaluate the mechanical behaviour of the composites in a hydrated environment. For this purpose, the specimens of each group were immersed in phosphate-buffered saline (PBS, pH 7.4) at 37 °C for 24 h to reach equilibrium moisture absorption before testing. The tests were then performed at 37 °C using a temperature-controlled environmental chamber (Model 3119-600, Instron, USA) integrated with the testing machine to maintain stable temperature and humidity throughout the measurement. After completing the mechanical tests (compression/tensile), specimens were photographed, and the fractured or deformed standard dry specimens were collected and immediately stored in a desiccator to prevent moisture absorption. Prior to SEM analysis, the specimens were sectioned using a precision saw to expose the fracture surface (for tensile tests) or the lateral crack propagation region (for compression tests), ensuring minimal mechanical damage to the target area. The SEM experimental conditions were kept consistent with those used for surface morphology observation. Multiple magnifications were used to observe fracture morphology, crack initiation and propagation patterns, filler–matrix interfacial bonding, and failure mechanisms.

#### 2.5.6 Thermal tests

Differential scanning calorimetry (DSC) analysis was performed using a DSC instrument (Q20, TA Instruments, USA) on 3D-printed disk-shaped specimens (diameter: 10 mm; height: 3 mm) under a nitrogen atmosphere. The samples were first equilibrated at −30 °C, then heated to 200 °C at a rate of 10 °C/min, held isothermally for 5 min, and subsequently cooled to −30 °C at the same rate. The heating/cooling cycles were repeated to record both melting and crystallization behaviours. The obtained DSC data were plotted using Origin 2024 and analysed with TA Advantage software to determine the glass transition temperature (T_g_), cold crystallization temperature (T_cc_), cold crystallization enthalpy (ΔH_cc_), melting temperature (Tm), and melting enthalpy (ΔH_m_). The degree of crystallinity (X_cc_) of pure PLA and composites was calculated according to the following equation:

$$Xcc=\frac{\Delta H_{m}-\Delta H_{CC}}{\frac{\Delta H_{m}^{0}}{wt\%\;of\;PLA}}$$

where ΔH_m_^0^ is the enthalpy of melting for 100% crystalline PLA, taken as 93 J/g.

#### 2.5.7 *In vitro* degradation behaviour test

Simulated body fluid (SBF; Solarbio, China) was used for the *in vitro* degradation experiment. 3D-printed scaffolds (13.6 mm × 3.2 mm, diameter × height) were prepared for each experimental group, with 6 samples per group. The scaffolds were accurately weighed (W_0_) and then immersed in 10 mL of SBF at 37 °C under 100 rpm agitation. The degradation test lasted for 12 weeks. Every 2 weeks, the samples from each group were removed from SBF, dried under vacuum at 37 °C for 24 h, weighed (W_1_), and the weight loss rate (WLR) was calculated using the equation:

$$WLR=\frac{W_{0}-W_{1}}{W_{0}}\times 100\%$$

### 2.6 Cell experiments

#### 2.6.1 Sterilization of cell cultures and materials

Rat bone marrow stem cells (rBMSCs) were used for cell experiments, cultured in α-MEM (Gibco, America) complete medium supplemented with 10% (v/v) fetal bovine serum (FBS, Gibco, America). The incubator conditions were set at 37 °C and 5% (v/v) CO_2_. When the cells reached 80% confluency in the culture plate, they were digested with 0.25% (w/w) trypsin (Gibco, America) and passaged at a 1:3 ratio. Cells were seeded for experiments from generation 3.

3D-printed cylindrical scaffold samples (10 mm × 3 mm, diameter × height) were fabricated via 3D printing. The samples were immersed in 75% ethanol three times (10 min each) and washed with PBS three times, followed by ultraviolet (UV) irradiation for 8 h (4 h per side) in a laminar flow clean bench for use.

#### 2.6.2 Cell proliferation activity test

rBMSCs were seeded onto scaffold samples at a density of 5 × 10^4^ cells/mL in 24-well plates. After 24-h culture in complete medium, when the cells had adhered, cells were further cultured for 3 days, and CCK-8 assays were conducted at 24, 48, and 72 h to assess cell proliferation. At each time point, 10% (v/v) prepared CCK-8 solution (CCK-8, Beyotime Biotechnology, Shanghai, China) was added to each well and incubated for 1.5 h. The absorbance at 450 nm (optical density, OD) was measured using a microplate reader (Synergy H1, Thermo Scientific, USA).

#### 2.6.3 Cell adhesion assay

Samples described above were placed in 24-well plates, and RBMSCs were seeded onto the material surfaces at a density of 1 × 105 cells/mL. After 24 h, the medium was removed, and the samples were rinsed three times with a sodium chloride-free phosphate buffer (PB) solution. Cells were fixed with 2.5%glutaraldehyde fixation solution (Solarbio, China) at room temperature for 20 min, followed by 3 PB washes. Dehydration was carried out using ethanol solutions of increasing concentrations (20%, 50%, 70%, 90%, and 100%) for 10 min each, followed by 2 times of 100% ethanol dehydration and tert-butanol substitution. Samples were pre-frozen at −80 °C for 2 h and then freeze-dried overnight. A layer of gold film of 15 nm thickness was sputtered on the surfaces of the dried samples, which were observed under an S-4800 SEM (RILI, China) at an accelerating voltage of 3 kV. Images were captured at appropriate magnifications with adjusted brightness.

#### 2.6.4 Cell live/dead staining and cytoskeleton staining

rBMSCs were seeded into 24-well Transwell lower chamber at a density of 5 × 10^4^ cells/mL. And scaffold samples were placed in the upper chamber. After 24-h co-culture, AM/PI staining solution was prepared by mixing 10 μL of Calcein-AM and 20 μL of PI dye in 5 mL of PBS. Each well received 200 μL of AM/PI staining solution and was incubated in the dark for 30 min. Live and dead cells were observed using an in-verted fluorescence microscope at excitation wavelengths of 490 nm and 535 nm, respectively. After 48-h co-culture, the medium was then discarded, and the cells were rinsed three times with PBS, followed by 4% PFA fixation at room temperature for 20 min. After that, the cells were rinsed and incubated with 0.1% Triton X-100 (Beyotime, China) at room temperature for 20 min. Actin filaments were stained using Ac-tin-Tracker Red-555 (Beyotime, China). The cells were covered by antifade mounting medium with DAPI (Beyotime, China), and images were captured under a fluorescence inverted microscope at ×200 magnification for observation.

#### 2.6.5 Alkaline phosphatase staining and alizarin red staining

rBMSCs were seeded into 24-well Transwell lower chamber at a density of 5 × 10^4^ cells/mL. And scaffold samples were placed in the upper chamber. The osteogenic in-duction medium was prepared by supplementing α-MEM with 10 mM β-glycerophosphate (Solarbio, China), 50 μg/mL ascorbic acid (vitamin C; Solarbio, China), 10 nM dexamethasone (Solarbio, China), and 2% (v/v) FBS (Gibco, USA). On day 7 of osteogenic induction, the medium was discarded, and the cells were washed three times with PBS, fixed in 4% PFA for 20 min. The Alkaline Phosphatase (ALP) Staining Kit (Beyotime, China) was used for staining according to the manufacturer’s instructions. The samples were incubated in dark at room temperature for 18 h, after which images were captured under a 100× microscope. Five fields of view were photographed, and ImageJ software was used to perform grayscale quantification analysis of the stained regions observed under the microscope. With the same osteogenic induction conditions, on day 14 and 28 of osteogenic induction, cells were rinsed and fixed, followed by 30-min Alizarin Red S Solution (Oricell, China) staining. After rinsing, images were captured under an inverted microscope (Zeiss, Germany) to evaluate the formation of calcium nodules. Subsequently, 10% cetylpyridinium chloride solution was added to each well and thoroughly mixed. The OD value of the supernatant was measured at 562 nm for quantitative analysis. All experiments were performed with three independent biological replicates (n = 3).

#### 2.6.6 Western blotting and quantitative real-time PCR analysis (qRT-PCR)

After 7-day osteogenic induction same as above, total protein from was extracted using radioimmunoprecipitation assay (RIPA) buffer. Protein concentration was deter-mined using the bicinchoninic acid (BCA) assay. Equal amounts of protein were subjected to sodium dodecyl sulfate–polyacrylamide gel electrophoresis (SDS-PAGE) and transferred onto pure nitrocellulose membranes. The membranes were blocked with 5% (w/v) skim milk at room temperature for 1 h, followed by overnight incubation at 4 °C with primary antibodies against BMP-2 (1:1000 dilution, ab179483, Abcam), OCN (1:1000 dilution, ab32152, Abcam), ALP (1:1000 dilution, ab229126, Abcam), and Runx2 (1:1000 dilution, ab92336, Abcam). After washing, appropriate horseradish peroxidase (HRP)-conjugated secondary antibodies (anti-rabbit and anti-mouse) were applied, and the immunoreactive bands were visualized using the Pro-light HRP detection kit.

Total RNA from rBMSCs in each group was extracted using TRIzol reagent. Relative gene expression was assessed by quantitative real-time PCR (qRT-PCR), with β-actin serving as the internal control. All PCR amplifications were performed in a final reaction volume of 20.0 μL using specific primer pairs listed in Table 1. The amplification was carried out for 40 cycles using Hieff qPCR SYBR Green Master Mix (Yeason, 11201ES03, China), and relative expression levels were calculated using the 2^–ΔΔCt^ method. Each assay was conducted in triplicate with three independent biological replicates (n = 3).

**TABLE 1:** Specific primers for immunoregulatory and osteogenesis-related genes.

Gene name	Forward primer sequence (5′-3′)	Reverse primer sequence (5′-3′)
β-actin	CCC​GCG​AGT​ACA​ACC​TTC​TT	CGC​AGC​GAT​ATC​GTC​ATC​CA
BMP2	TGGACTTCAGTGATGTGG	ATGGTTGGTGGAGTTCAG
RUNX2	TTCGTCAGCGTCCTATCA	CAGCGTCAACACCATCAT
Osx	AGGAAGAAGCCCATTCAC	GAACCTCTTGCCACAGAA
COL1A1	CTGTGCCTCAGAAGAACT	ACCTTCGCTTCCATACTC

### 2.7 *In vivo* experiments

#### 2.7.1 Establishment of SD rat cranial defect model

To evaluate the early-stage osteogenic properties of 3D-printed bone scaffolds, 15 SD rats (male, 10 weeks old, average weight 250 g) were randomly divided into three groups: the control group (no implantation, n = 5), the PLA group (0wt% TCP, n = 5) and the 80:20 group (20wt% TCP, n = 5). The animal experiment protocol was approved by the Animal Welfare and Ethics Committee of Capital Medical University, No. KQYY-201912-002. The surgeries were performed under sterile conditions. Anesthesia was induced with 5% isoflurane and maintained with 2% isoflurane using a calibrated vaporizer with continuous oxygen flow. Bilateral full-thickness circular bone defects (6 mm in diameter) were created along the cranial suture using a trephine drill, with continuous saline irrigation for cooling. Sterilized cylindrical bone scaffolds (6 mm × 1 mm, diameter × height) were implanted into the bone defect sites. The wounds were sutured in layers using 4-0 absorbable sutures. Postoperatively, penicillin (10,000 U/day) was administered via intramuscular injection for 3 days.

#### 2.7.2 Postoperative observation

After recovery from anesthesia, the rats were maintained under the same housing conditions as before surgery. Postoperative observations included general condition, food intake, activity levels, and the status of the surgical incision.

#### 2.7.3 Micro-CT analysis

At 4 weeks post-implantation, rats were deeply anesthetized with 5% isoflurane delivered by inhalation in oxygen until loss of righting reflex (LORR). Euthanasia was then performed by gradual-fill exposure to 100% CO_2_ at approximately 30% of the chamber volume per minute, continuing until respiratory and cardiac arrest were confirmed. The skulls were then collected and immediately fixed in 4% paraformaldehyde (PFA) for subsequent radiological and histological evaluations. The fixed cranial specimens of rats were scanned using Micro-CT to acquire cross-sectional images in the axial, coronal, and sagittal planes of the entire skull. Regions of interest (ROIs) were then defined at the defect sites. Using Micro-CT software, both qualitative and quantitative analyses (bone volume to total volume ratio, BV/TV) of new bone formation were performed.

#### 2.7.4 Preparation of paraffin-embedded tissue sections

The fixed cranial specimens of rats were removed from 4% PFA and rinsed under running water for 30 min to thoroughly eliminate residual fixative. Subsequently, the cleaned cranial samples were immersed in a 10% EDTA solution for decalcification over a period of 6 weeks. After decalcification, the cranial samples were soaked in sterile PBS for 12 h, followed by another 30–35 min of rinsing under running water. The bone specimens were then trimmed using a surgical scalpel and fine scissors to remove surrounding soft tissues. Dehydration was carried out sequentially in graded ethanol solutions (80%, 90%, 95%, and 100%), with each step lasting 2 h. After dehydration, the samples were immersed in xylene for 3 h for clearing. At last, the bone samples were embedded by immersion in molten paraffin at 55 °C for 1.5 h. The molten paraffin was poured into molds, and the specimens were carefully positioned in the molds using pre-warmed forceps. After paraffin embedding, the condylar bone tissue samples were sectioned at a thickness of 5 μm. Sections were picked up on clean glass slides, and then further spread using a slide spreader. The slides were briefly air-dried at room temperature and subsequently baked in a 60 °C oven for 4 h to remove residual moisture. The prepared tissue sections were then stored for further use.

#### 2.7.5 H&E staining

The sections were routinely dewaxed and immersed in hematoxylin solution for 6 min. Afterwards, they were gently rinsed under running water for 8 min to remove excess stain from the slides. The sections were then differentiated in 1% acid alcohol solution until the nuclei appeared blue under a microscope. Following this, the slides were rinsed again under running water for 10 min and subsequently immersed in 0.5% eosin solution for 2 min for counterstaining. The stained sections were de-hydrated in a graded ethanol series (75%, 80%, 90%, 95%, and 100%) for a total of 30 min, followed by two rounds of clearing in xylene for approximately 5 min each. Finally, the sections were mounted using neutral resin at room temperature and allowed to dry in a dark, ventilated area. Once dry, the slides were observed and imaged using a slide scanning imaging system (Shengqiang Technology, China).

#### 2.7.6 Masson staining

The dewaxing and hematoxylin staining procedures were the same as those used in H&E staining. Masson’s Ponceau-Fuchsin solution was then applied to stain the sections for 15 min, then briefly rinsed with weakly acidic water for 30 s. Sections were treated with phosphomolybdic acid solution for 10 min for differentiation, stained with aniline blue solution for 5 min, and again rinsed gently with weak acid water for 30 s. The subsequent dehydration, clearing, and observation procedures were the same as those used in H&E staining.

### 2.8 Data analysis

Statistical analyses and plotting were performed using Origin 2024 and GraphPad Prism 10.0 software. One-way analysis of variance (ANOVA) was used to assess statistical significance, and results were expressed as mean ± standard deviation (*x̅* ± s). *P < 0.05, **P < 0.01, ***P < 0.001, ****P < 0.0001 The difference is statistically significant.

## 3 Results

### 3.1 Material characterization

#### 3.1.1 Particle size and mixing state

**FIGURE 2:**
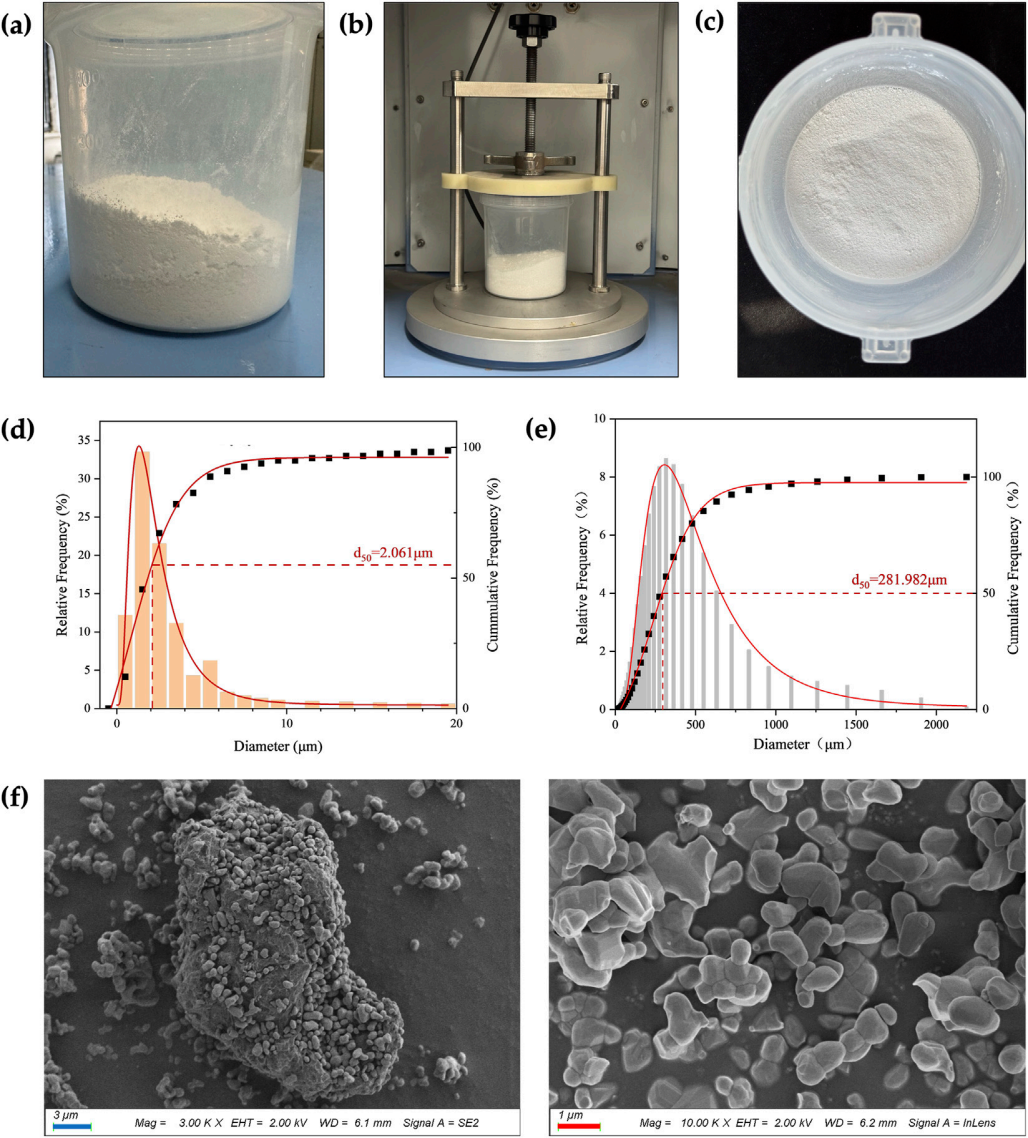
Particle size and mixing state. **(a)** The PLA powder (bottom layer) and β-TCP powder (top layer) were layered separately before RAM. **(b)** During RAM. **(c)** The composite powder exhibited a homogeneous state after RAM. **(d)** β-TCP particle size analysis. **(e)** PLA particle size analysis. **(f)** SEM images of the composite powder. (blue bar = 3 μm, red bar = 1 μm).

Prior to mixing, the PLA and β-TCP powders appeared stratified, as shown in Figure 2a. After the RAM procedure (Figure 2b), the composite powders became fully blended without visible stratification, forming a homogeneous mixture, as Figure 2c exhibits. The particle size analysis, as can be seen in Figures 2d,e, showed that the β-TCP powder exhibited a uniform overall particle size distribution, with a d_50_ of 2.061 μm, which was significantly smaller than that of the PLA powder (d_50_ = 281.982 μm). The favorable particle size of β-TCP allowed it to be evenly dispersed within the PLA matrix during the RAM process as a guest powder. SEM images, as shown in Figure 2f, further revealed that the fine β-TCP particles adhered to the surface of the larger PLA particles, with some even partially embedded into the PLA particles.

#### 3.1.2 Phase characterization and WCA measurement

**FIGURE 3:**
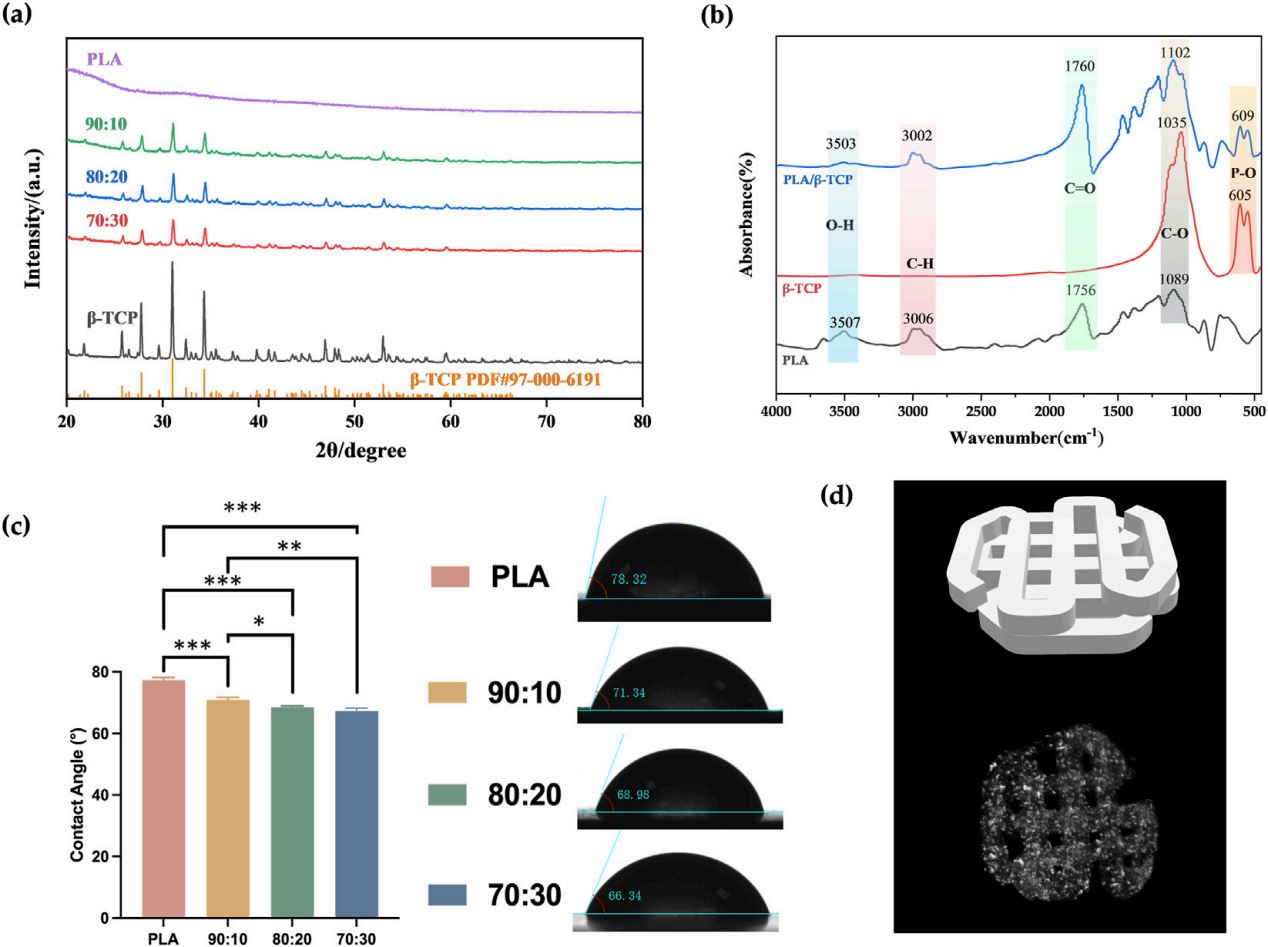
Phase characterization and WCA measurement. **(a)** XRD patterns of PLA alone and with different β-TCP proportions (PLA: β-TCP = 90:10, 80:20, 70:30). **(b)** FTIR spectra of PLA, β-TCP, and PLA/β-TCP. **(c)** WCA of PLA alone and with different β-TCP proportions. **(d)** Three-dimensional visualization of the 3D-printed scaffold: (Top) STL model illustrating the designed pore geometry and lattice architecture. (Bottom) Micro-CT reconstruction of the PLA/β-TCP scaffold (80:20).

Phase characterization of β-TCP, PLA, and composite powders with varying ratios was conducted using XRD. As shown in Figure 3a, the characteristic peaks observed at 2θ values of 27.78°, 31.02°, and 34.33° corresponded well with the standard β-TCP structure (JCPDS card #97-000-6191). PLA, being amorphous, exhibited no distinct diffraction peaks in the XRD analysis. No impurity crystalline phases other than β-TCP were detected, and all PLA/β-TCP composite powders showed consistent diffraction patterns, confirming that β-TCP was the sole inorganic component.

FTIR was used to analyze the functional groups of the synthesized powders. As shown in Figure 3b, peaks at 605.5 cm^-1^ and 551.6 cm^-1^ in the FTIR spectrum of β-TCP corresponded to P–O and Ca–O stretching vibrations from phosphate groups, confirming the presence of calcium and phosphate. In the PLA spectrum, the peak at 1085.7 cm^-1^ corresponded to C–O stretching, while the peak at 2996.9 cm^-1^ was attributed to C–H stretching from methyl (–CH_3_) groups. The peak at 1758.8 cm^-1^ represented the ester carbonyl (C=O) stretch. The PLA/β-TCP composites exhibited both the characteristic PLA absorption bands and the PO_4_^3-^ bands, confirming the presence of both components.

WCA measurements, as can be seen in Figure 3c, showed that pure PLA samples had a contact angle of 77.33° ± 0.73°, which was significantly higher than that of the β-TCP-containing groups. As the β-TCP content increased from 10% to 20%–30%, the surface roughness increased, and the contact angle correspondingly decreased from 70.90° ± 0.65° to 68.48° ± 0.39°, and finally to 67.33° ± 0.70°. And micro-CT visualization (Figure 3d) confirmed that the printed PLA/β-TCP scaffold reproduced the designed STL lattice with continuous interconnected pores. The internal gray-scale distribution indicated homogeneous ceramic incorporation and high structural fidelity.

#### 3.1.3 Surface morphology of filaments and 3D-printed scaffolds

**FIGURE 4:**
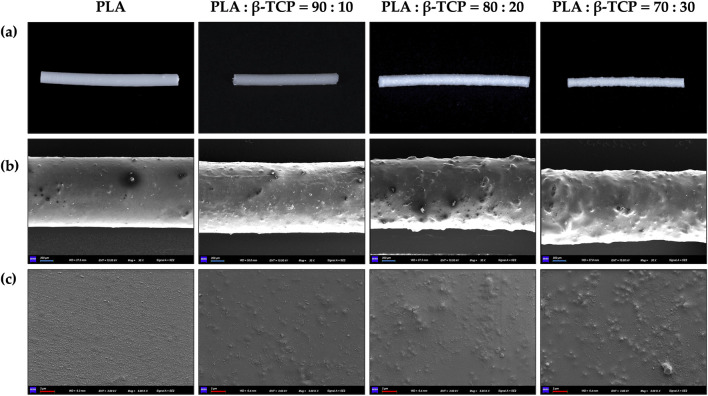
Macroscopic and microscopic morphology of the extruded filaments of PLA alone and with different β-TCP proportions. **(a)** Photographic images of the filaments, respectively. **(b)** SEM images of the filaments at ×30 magnification, respectively. **(c)** SEM images of the filaments at 5k × magnification, respectively. (blue bar = 300 μm, red bar = 2 μm).

Macroscopic photographs and SEM revealed that the surface of the pure PLA filaments was smooth and free of noticeable protrusions, as shown in Figure 4. As the β-TCP content increased, the surface roughness of the filaments also increased.

**FIGURE 5:**
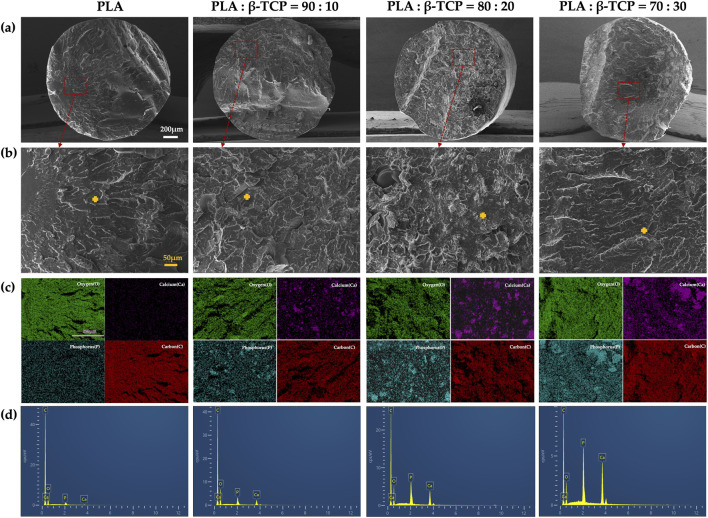
Cross-sectional morphology and elemental analysis of extruded PLA filaments with different β-TCP proportions: **(a)** SEM images of the liquid-nitrogen freeze-fractured cross sections at low magnification (×55) for each group (white bar = 200 μm). **(b)** SEM images of the corresponding regions (200×) (orange bar = 50 μm), with orange crosses indicating the sites selected for EDS point analysis. **(c)** EDS elemental mapping images (C, O, Ca, P) of representative areas (pueple bar = 250 μm). **(d)** EDS point spectra acquired from the marked positions.

The liquid nitrogen freeze-fractured cross-sections of the filaments further revealed the distribution characteristics of β-TCP within the PLA matrix (Figure 5). In the 90:10 and 80:20 groups, ceramic particles were finely and uniformly dispersed throughout the polymer phase, with no evident pores or phase separation, indicating good interfacial compatibility and favourable load transfer. The corresponding EDS elemental (Ca, P) maps further confirmed this homogeneous spatial distribution, suggesting that β-TCP was well embedded within the filament cross-sections. In contrast, when the β-TCP content reached 30 wt% (70:30 group), locally aggregated particles and denser Ca/P-rich regions were observed, indicating more pronounced particle agglomeration during filament extrusion. EDS point spectra acquired from the marked regions showed significantly increased Ca and P peak intensities with higher β-TCP contents, while the pure PLA group exhibited only background-level signals.

**FIGURE 6:**
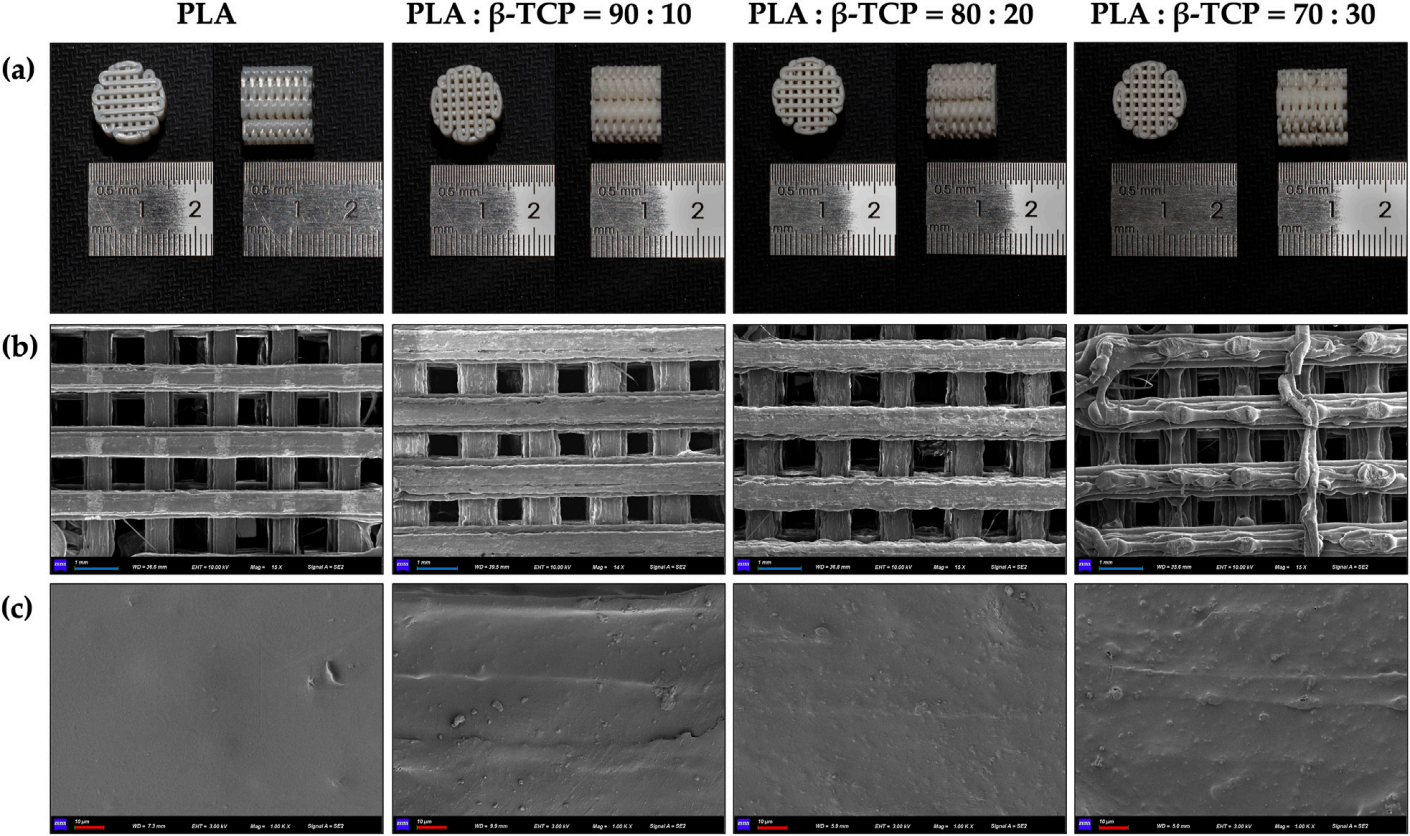
Macroscopic and microscopic morphology of the 3D-printed scaffolds of PLA alone and with different β-TCP proportions. **(a)** Photographic images of the 3D-printed scaffolds, respectively. **(b)** SEM images of the 3D-printed scaffolds at ×15 magnification, respectively. **(c)** SEM images of the 3D-printed scaffolds at 1k × magnification, respectively. (blue bar = 1 mm, red bar = 10 μm).

The surface microstructure of the 3D-printed scaffolds was generally consistent with that of the corresponding filaments, as can be seen in Figure 6, showing a clear interlaced porous architecture. However, the increasing proportion of β-TCP led to the agglomeration of surface particles, forming protruding structures on the filaments. This effect was particularly pronounced in the 70:30 group, where the difficulty in maintaining uniform filament diameter resulted in inconsistent extrusion flow during FDM printing, thereby affecting the overall forming quality of the printed scaffolds.

### 3.2 Mechanical tests

**FIGURE 7:**
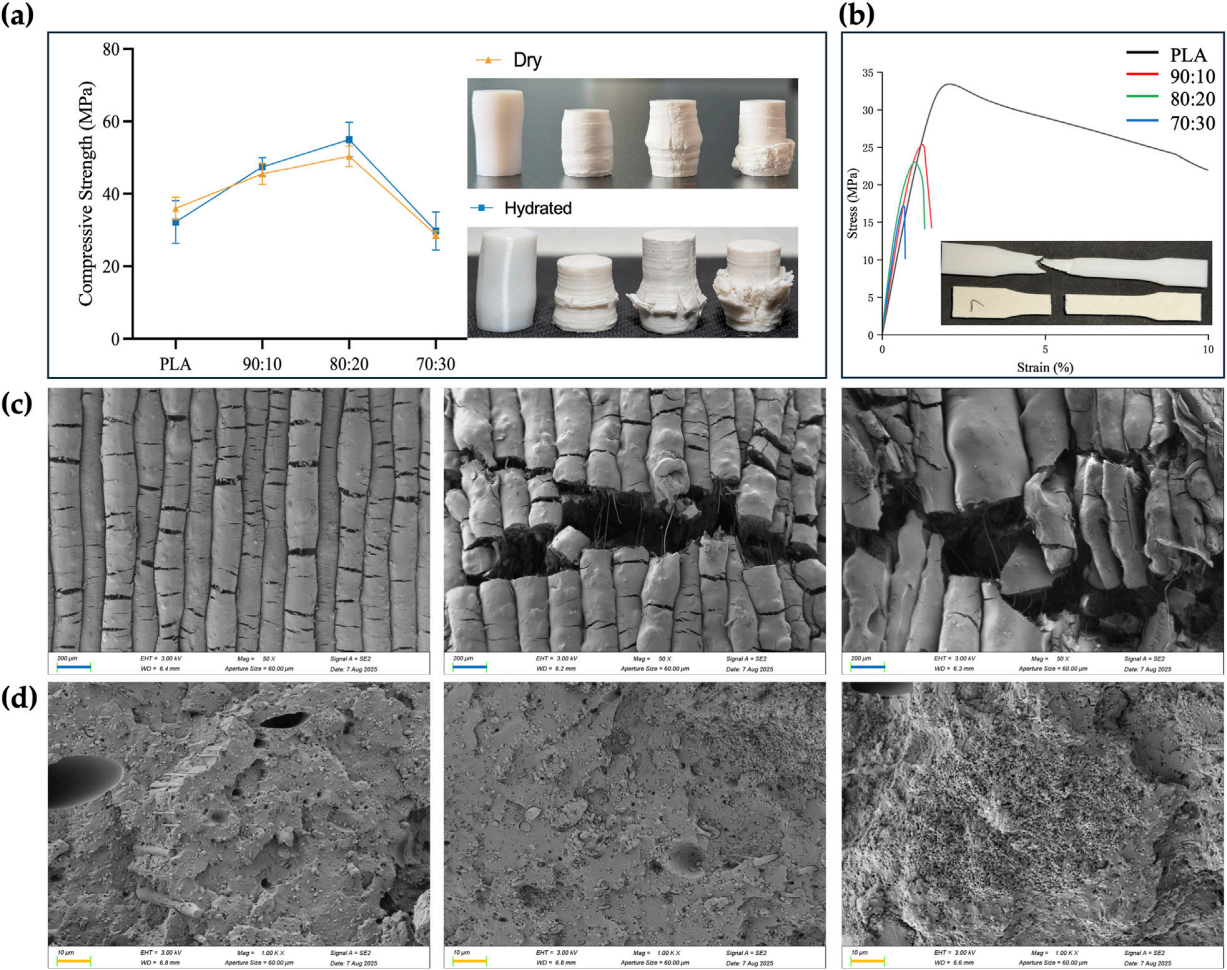
Mechanical properties, post-test photographs and SEM images of the samples. **(a)** Compressive strength of PLA and PLA/β-TCP composites under dry and hydrated conditions (ASTM D695-10, mean ± SD, n = 3), and representative post-compression specimens (left to right: PLA, 90:10, 80:20, 70:30). **(b)** Stress–strain curves from tensile tests (ASTM D638, n = 3), representative post-tensile fracture morphologies of PLA (top) and PLA/β-TCP (bottom) specimens are shown. **(c)** SEM images (50×) of compressed dry specimen surfaces showing crack propagation patterns for PLA/β-TCP composites with different compositions, from left to right: 90:10, 80:20, and 70:30. **(d)** SEM images (1000×) of tensile fracture surfaces for PLA/β-TCP composites with different compositions, from left to right: 90:10, 80:20, and 70:30. (blue bar = 200 μm, orange bar = 10 μm).

Mechanical testing was performed on standard specimens prepared according to ASTM D695-10 for compression and ASTM D638-14 for tension. Tests were conducted at a loading rate of 1.0 mm/min (n = 3), and mechanical parameters were calculated from the stress–strain curves (Table 2). In both dry and hydrated environments, the compressive strength initially increased with β-TCP incorporation and reached a maximum at the 80:20 composition, followed by a sharp decline when the β-TCP content was further increased to 30 wt%. Correspondingly, progressive surface cracking and more severe deformation were observed at higher β-TCP loadings (Figure 7a). Interestingly, hydrated pure PLA specimens exhibited slightly lower compressive strength compared to their dry counterparts, whereas β-TCP-containing samples showed a slight increase after hydration. SEM images (Figure 7c) revealed that the 90:10 group presented fewer cracks, mostly regular and perpendicular to the printed deposition layers, with uniform spacing, narrow widths, and smooth fracture surfaces without apparent delamination or fiber pullout traces. In contrast, the 80:20 group exhibited more numerous cracks, some of which crossed and connected between adjacent deposition layers, along with localized delamination and edge burrs, indicating microscale shear and fracture propagation during compression. The 70:30 group displayed extensive penetrating cracks and pronounced material rupture.

**TABLE 2:** Mechanical properties of each group of samples. Compressive strength was calculated from compression test with three replicates, while Young’s modulus, tensile strength, and elongation at break were calculated from tensile test with three replicates.

Group	Compressive strength (MPa)		Young’s modulus (MPa)	Tensile strength (MPa)	Elongation at break (%)
	Dry	Hydrated			
PLA	35.9716 ± 3.0700	32.2027 ± 4.8922	22.6792 ± 1.0008	34.3716 ± 1.0100	21.8629 ± 1.8472
90:10	45.5232 ± 3.9460	47.4777 ± 2.5487	34.3716 ± 1.0100	27.2136 ± 1.8268	1.3497 ± 0.0650
80:20	50.4048 ± 2.6704	55.0008 ± 4.7852	21.8629 ± 1.8472	23.2497 ± 3.3855	1.0701 ± 0.1220
70:30	28.6986 ± 1.6014	29.7081 ± 4.2655	27.6630 ± 0.8947	17.1461 ± 1.8581	0.6695 ± 0.0316

The addition of β-TCP caused a sharp reduction in tensile performance, with the tensile fracture strain of the 70:30 group dropping to as low as 0.6% (Figure 7b). Pure PLA specimens exhibited good ductility in tensile testing, with filamentous structures visible at the fracture ends. In contrast, the incorporation of β-TCP particles markedly reduced tensile resistance, resulting in neat fracture surfaces with almost no plastic deformation of the internal material (Figure 7b). SEM images (Figure 7d) of the 70:30 tensile specimens revealed abundant β-TCP particles, pronounced particle agglomeration, and significant interfacial debonding, in stark contrast to the microstructural features observed in the 90:10 and 80:20 groups.

### 3.3 Calorimetric analysis

**FIGURE 8:**
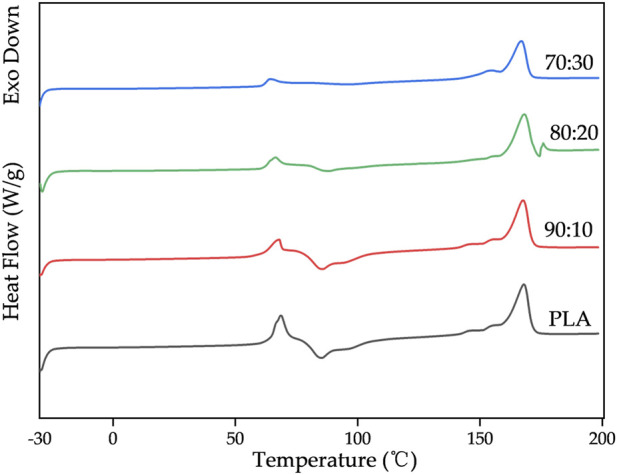
DSC thermograms of pure PLA and its composites.

Figure 8 shows the DSC thermograms of first and second heating run for pure PLA and its composites. The resultant thermal properties (Tg, Tcc, Tm, ΔHm, ΔHcc) obtained from the first heating run are summarized in Tables 3.

**TABLE 3:** Thermal properties and crystallinity fraction (Xcc) of pure PLA and its composites.

Group	T_g_ (°C)	T_cc_(°C)	ΔH_cc_ (J/g)	T_m_ (°C)	ΔH_m_ (J/g)	X_cc_ (%)
PLA	65.95	78.07	27.02	168.00	31.95	5.30
90:10	64.65	77.89	26.50	167.68	32.72	6.02
80:20	63.79	91.20	9.65	168.14	19.58	8.54
70:30	63.03	96.83	3.21	166.91	23.75	15.46

DSC results showed that the glass transition temperature (Tg) of PLA/β-TCP composites decreased slightly with increasing β-TCP content (from 65.95 °C to 63.03 °C), indicating that β-TCP slightly enhanced the mobility of PLA chain segments. In contrast, the cold crystallization behavior exhibited pronounced differences. At low β-TCP contents (0%–10%), the cold crystallization peak temperature (Tcc) remained stable at approximately 78 °C, with relatively high cold crystallization enthalpy (ΔHcc) values (26.50–27.02 J/g). When the β-TCP content increased to 20%–30%, Tcc rose markedly to 91.20 °C–96.83 °C, while ΔHcc sharply decreased to 9.65–3.21J/g, suggesting that high β-TCP loading may severely inhibit cold crystallization kinetics via a steric hindrance effect. The melting peak temperature (Tm) remained essentially unchanged (166.91 °C–168.00 °C), whereas the melting enthalpy (ΔHm) displayed an increase trend, closely associated with changes in crystallinity (Xcc). Xcc increased significantly with higher β-TCP content, reaching 15.46% at 30% β-TCP, revealing that β-TCP may act as a heterogeneous nucleating agent to effectively promote PLA crystallization during processing. The discrepancy between the high Xcc and low ΔHcc values suggests that the crystallization was primarily induced during initial processing rather than through cold crystallization during testing.

### 3.4 *In vitro* scaffold degradation test

**FIGURE 9:**
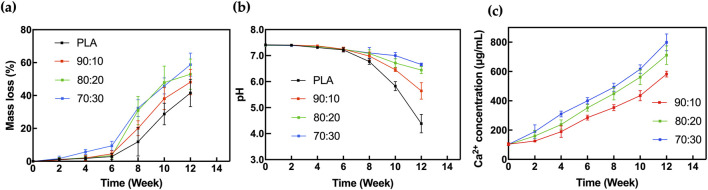
*In vitro* degradation analysis of **(a)** Mass loss, **(b)** pH change, **(c)** Calcium ion concentration for 3D-printed scaffolds in SBF.

The results of the degradation test showed that weight loss (Figure 9a) and pH change (Figure 9b) of the scaffolds were relatively slow during the first 6 weeks but increased significantly thereafter. The rapid mass loss phase primarily occurred after week 6, and the degradation rate accelerated with higher β-TCP content in the samples. Although early changes in weight loss and pH were not significant, the calcium ion concentration in the SBF solution (Figure 9c), showed marked differences as early as 2 weeks.

### 3.5 Early-stage cell adhesion and proliferation

**FIGURE 10:**
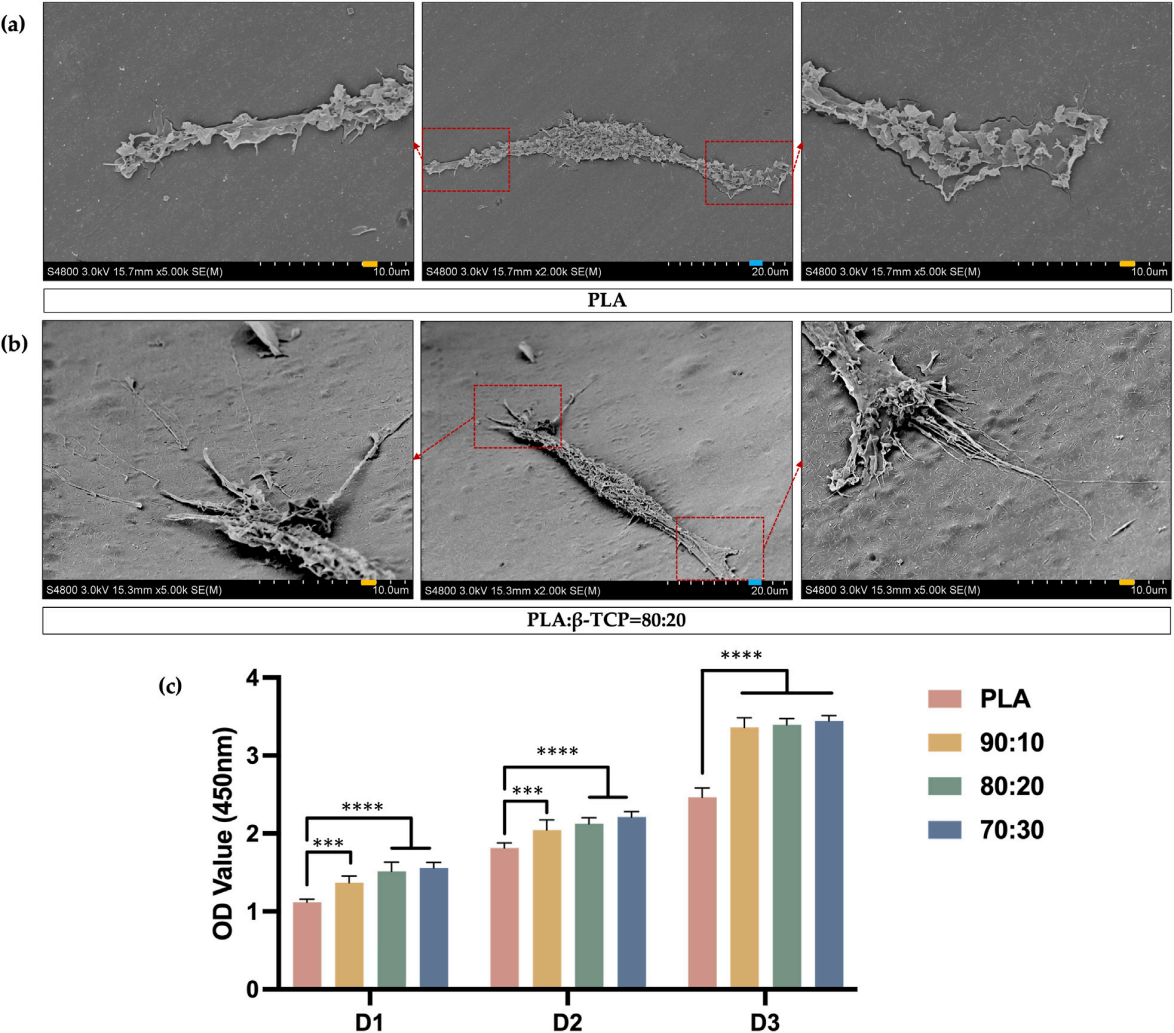
SEM images of cell adhesion and CCK-8 assay results. **(a)** SEM images of the adhesion morphology of rBMSCs seeded on the surface of PLA scaffolds. (blue bar = 20 μm, yellow bar = 10 μm). **(b)** SEM images of the adhesion morphology of rBMSCs seeded on the surface of PLA/β-TCP (80:20) scaffolds. **(c)** CCK-8 assays for days 1, 2, and 3 after seeding rBMSCs on the surfaces of different scaffolds.

The results of the cell adhesion experiment demonstrated the morphology of rBMSCs after 24 h of seeding on the sample surfaces, followed by fixation, graded dehydration, and freeze-drying. Cells were observed adhering to both the smooth PLA scaffold surfaces (Figure 10a) and the rough, protruded surfaces of the PLA/β-TCP composite scaffolds (Figure 10b). On the PLA/β-TCP group samples, rBMSCs exhibited excellent adhesion and spreading. Under SEM, numerous filopodia-like protrusions were visible around the cells, extending like tentacles and firmly anchoring to the scaffold surface. These elongated extensions indicated active cytoskeletal remodeling, suggesting that the PLA/β-TCP scaffolds provided a more favorable environment for early cell adhesion.

As is shown in Figure 10c, the CCK-8 assay results of rBMSCs cultured on different biomimetic bone scaffolds further confirmed that all scaffold groups supported good cell proliferation, indicating no significant cytotoxicity and good biocompatibility. Moreover, the scaffolds containing β-TCP showed enhanced cell proliferation activity compared to the pure PLA group.

Before 6 weeks of immersion in SBF, the pH of the solution exhibited a slight downward trend. However, there was no significant variation in pH among the groups during this period, and the SBF environment remained mildly alkaline. After 6 weeks, the degradation rate of PLA increased significantly. Since lactic acid is the primary degradation product of PLA, the solution’s pH dropped noticeably. In contrast, due to the buffering effect of β-TCP, the acidification in the PLA/β-TCP composite scaffold groups was significantly attenuated.

### 3.6 *In vitro* scaffold cytocompatibility test

**FIGURE 11:**
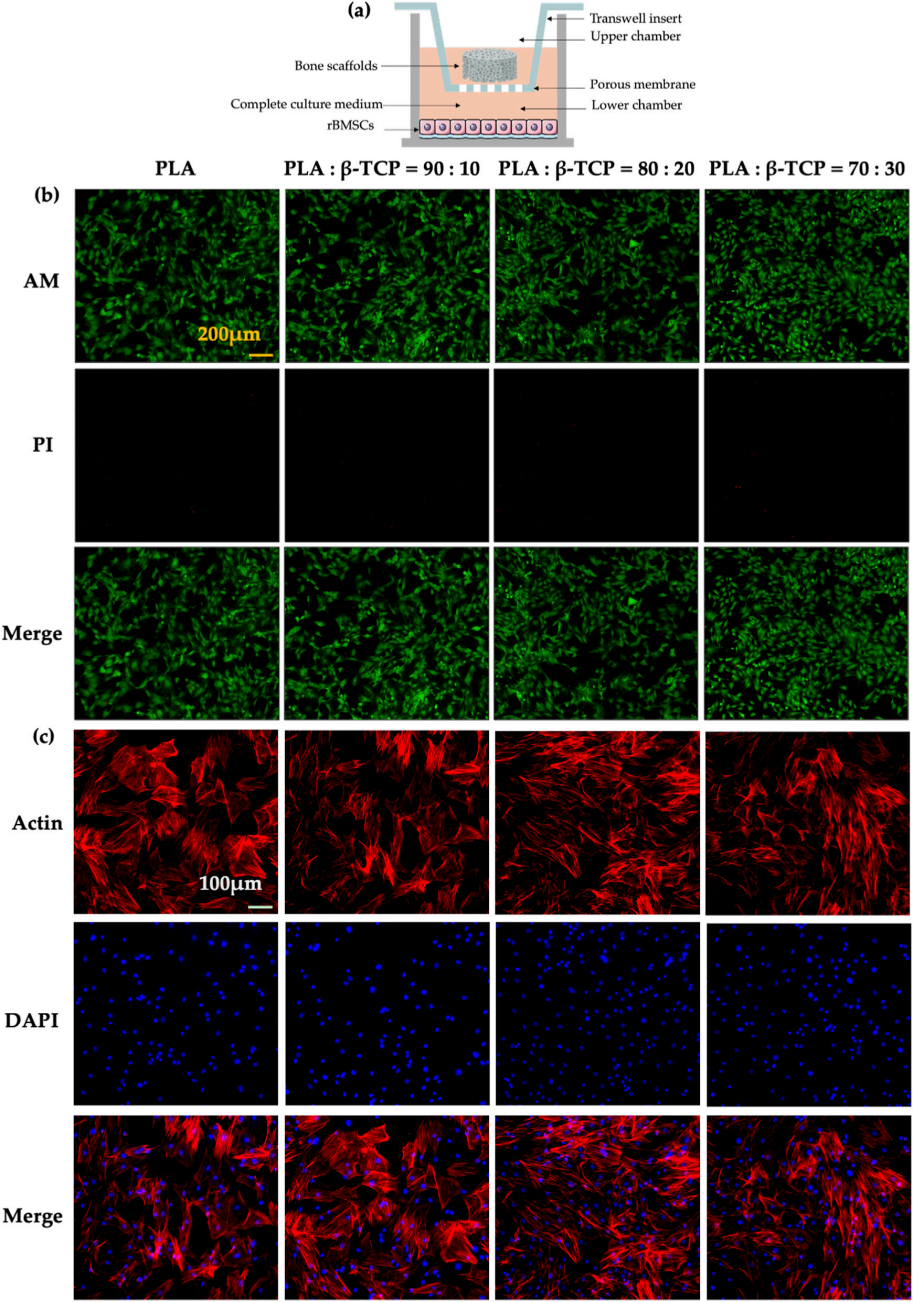
Biocompatibility of the scaffolds *in vitro*. **(a)** Schematic of the co-culture setup showing different scaffolds and rBMSCs in Transwell chambers. **(b)** Fluorescence images of rBMSCs stained with Calcein-AM and propidium iodide (PI) after 24 h of co-culture. (yellow bar = 200 μm). **(c)** Fluorescence images of cytoskeletal staining with phalloidin (Actin) and DAPI after 48 h of co-culture (white bar = 100 μm).

The *in vitro* biocompatibility of the scaffolds was evaluated using a Transwell co-culture system (Figure 11a), in which rBMSCs were seeded in the lower chambers, while different scaffold samples were placed in the upper inserts. Live/dead cell staining results (Figure 11b) showed that the cells in all groups exhibited healthy morphology with almost no red fluorescent dead cells, indicating good biocompatibility of all scaffold groups. Cytoskeletal fluorescence images (Figure 11c) revealed the morphology of rBMSCs co-cultured on scaffolds from different groups. The cells in all groups displayed healthy shapes and well-distributed growth. In the β-TCP-containing groups, especially the 80:20 and 70:30 groups, cells exhibited dense and confluent multilayered morphologies with clearly interconnected actin filaments.

### 3.7 *In vitro* scaffold osteogenic performance assessment

To evaluate the osteogenic performance of the biomimetic bone scaffolds, we co-cultured rBMSCs with each scaffold group to assess their osteogenic differentiation potential. We used alkaline phosphatase (ALP) activity and calcium nodule deposition as early and late markers of osteogenic differentiation, respectively.

**FIGURE 12:**
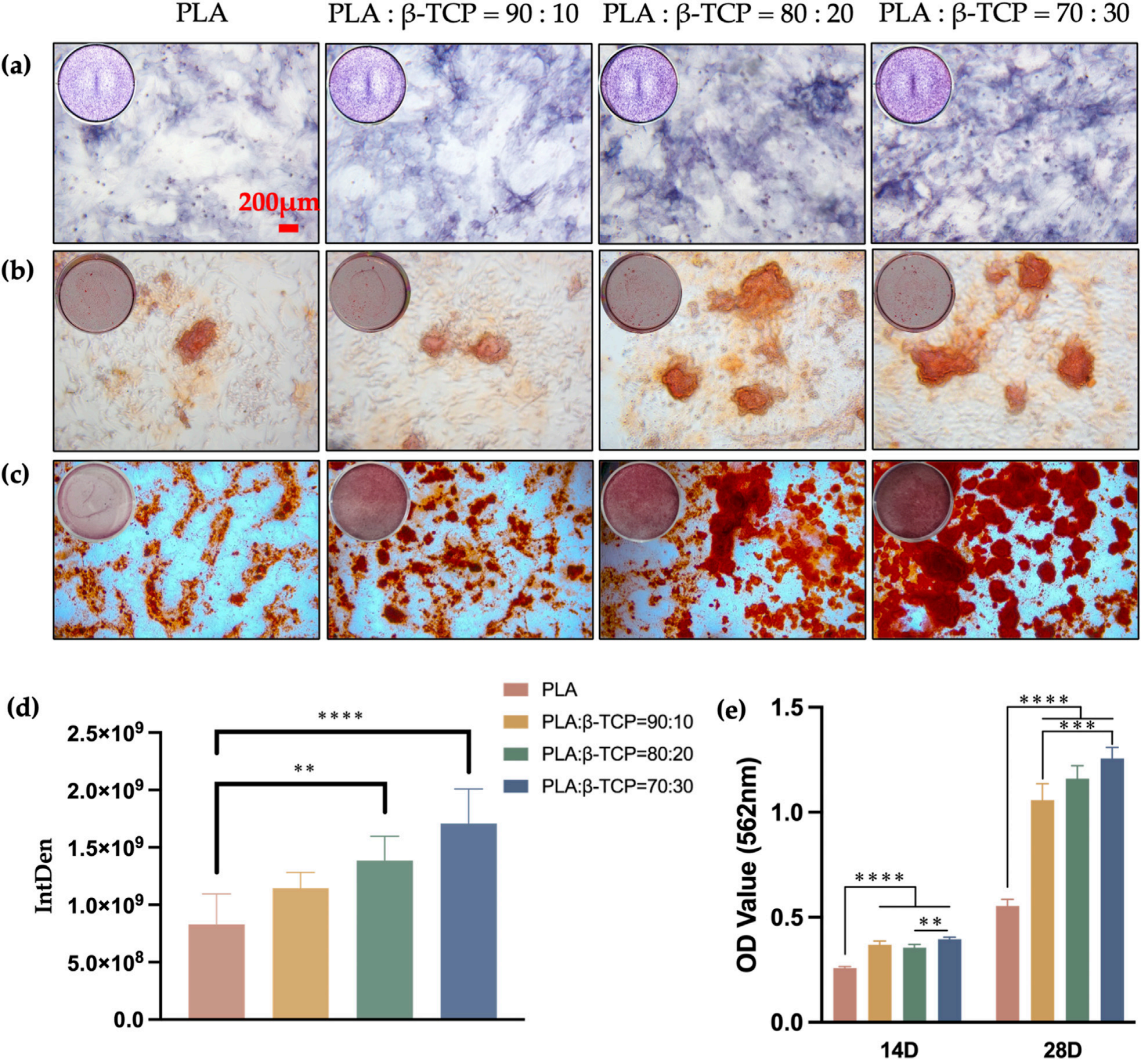
Osteogenic differentiation of the scaffolds *in vitro*. The qualitative assay of ALP, 7 days **(a)** and ARS, 14 **(b)**, 28 **(c)** days (red bar = 200 μm). **(d)** The quantitative assay of ALP, 7 days **(e)** The quantitative assay of ARS, 14, 28 days.

After 7 days, ALP staining (Figure 12a) revealed that rBMSCs co-cultured with β-TCP-containing scaffolds exhibited significantly higher ALP expression compared to the PLA group. Furthermore, ALP staining intensity increased with higher β-TCP content, with statistically significant differences observed between the 80:20 and 70:30 groups and the PLA group.

While Alizarin Red staining (ARS) at 14 days (Figure 12b) did not show significant statistical differences between groups, the results at 28 days (Figure 12c) indicated that β-TCP-containing groups displayed higher red staining intensity and more mineralized calcium nodules. Quantitative analysis (Figures 12d,e) corroborated these staining results, showing a statistically significant difference in calcium nodule expression between the β-TCP-containing groups and the PLA group (p < 0.0001). This suggests that the incorporation of β-TCP not only promotes the expression of early osteogenic differentiation markers, such as ALP, but also enhances late-stage calcification capabilities.

**FIGURE 13:**
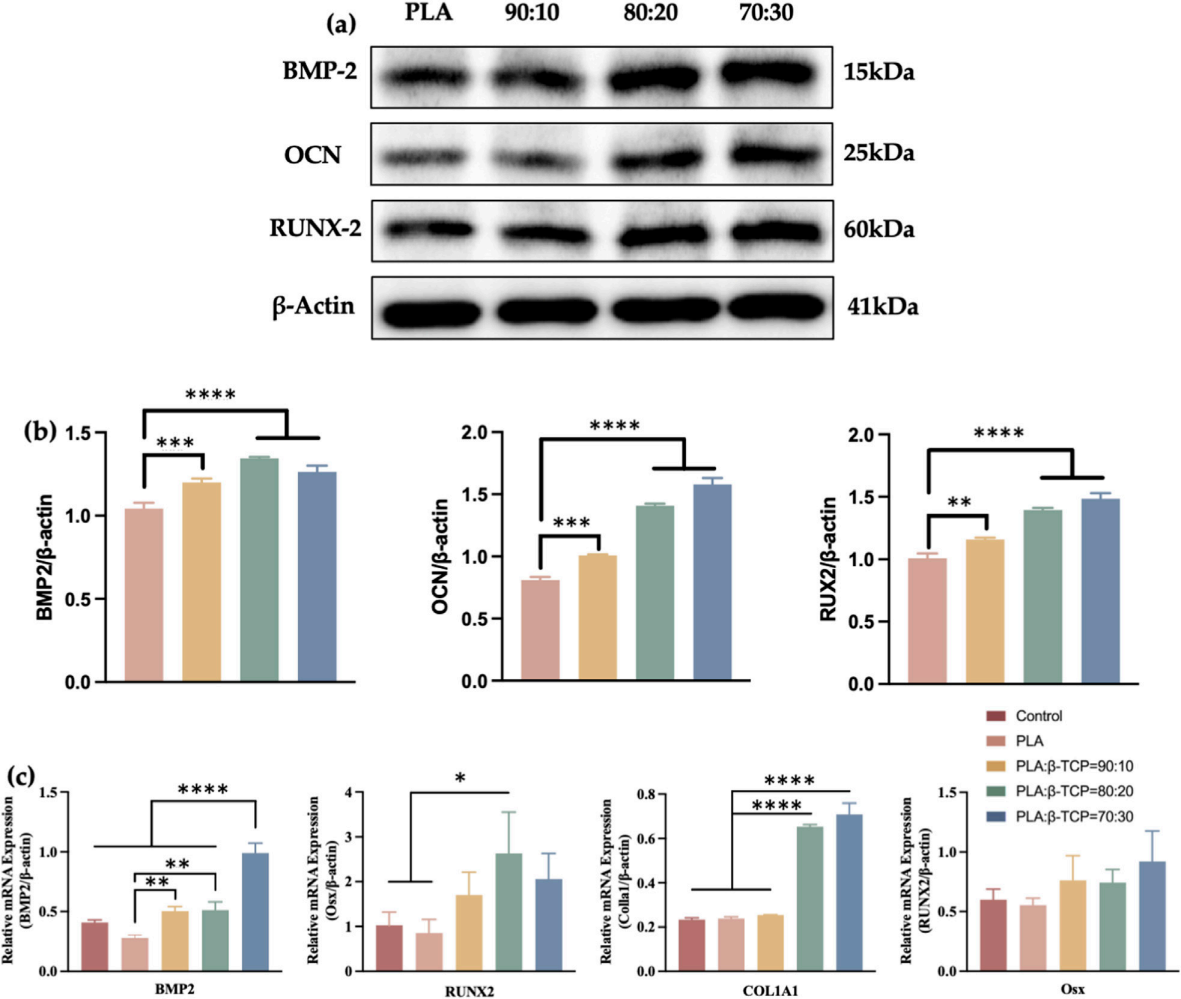
Transcriptomics analysis and Western blotting of the scaffolds. **(a)** The osteogenesis-related proteins of Western blot of pure PLA and groups with different β-TCP proportions. **(b)** The corresponding quantitative analysis of osteogenesis-related proteins. **(c)** Quantitative analysis of osteogenesis-related mRNAs.

This study also systematically evaluated the effects of composite scaffolds with var-ying β-TCP content on the protein and gene expression associated with osteogenic differentiation of rBMSCs. Western blot analysis (Figures 13a,b) revealed that, compared to the PLA control group, the composite scaffold groups exhibited significantly upregulated expression of RUNX2, osteocalcin (OCN), and BMP-2, particularly in the 70:30 and 80:20 β-TCP/PLA groups. β-Actin was used as the internal control and showed consistent ex-pression across all groups.

The relative expression levels of osteogenesis-related genes were also assessed by qRT-PCR after 7 days of osteogenic induction. As can be seen in Figure 13c, co-culturing with the composite scaffolds for 7 days notably enhanced the expression of Runx2 protein in rBMSCs, as well as the mRNA expression of COL1A1 and Runx2, especially in the 80:20 and 70:30 groups. However, Osx did not show statistically significant differences among the groups after 7 days of induction.

### 3.8 *In vivo* scaffold osteogenic performance assessment

**FIGURE 14:**
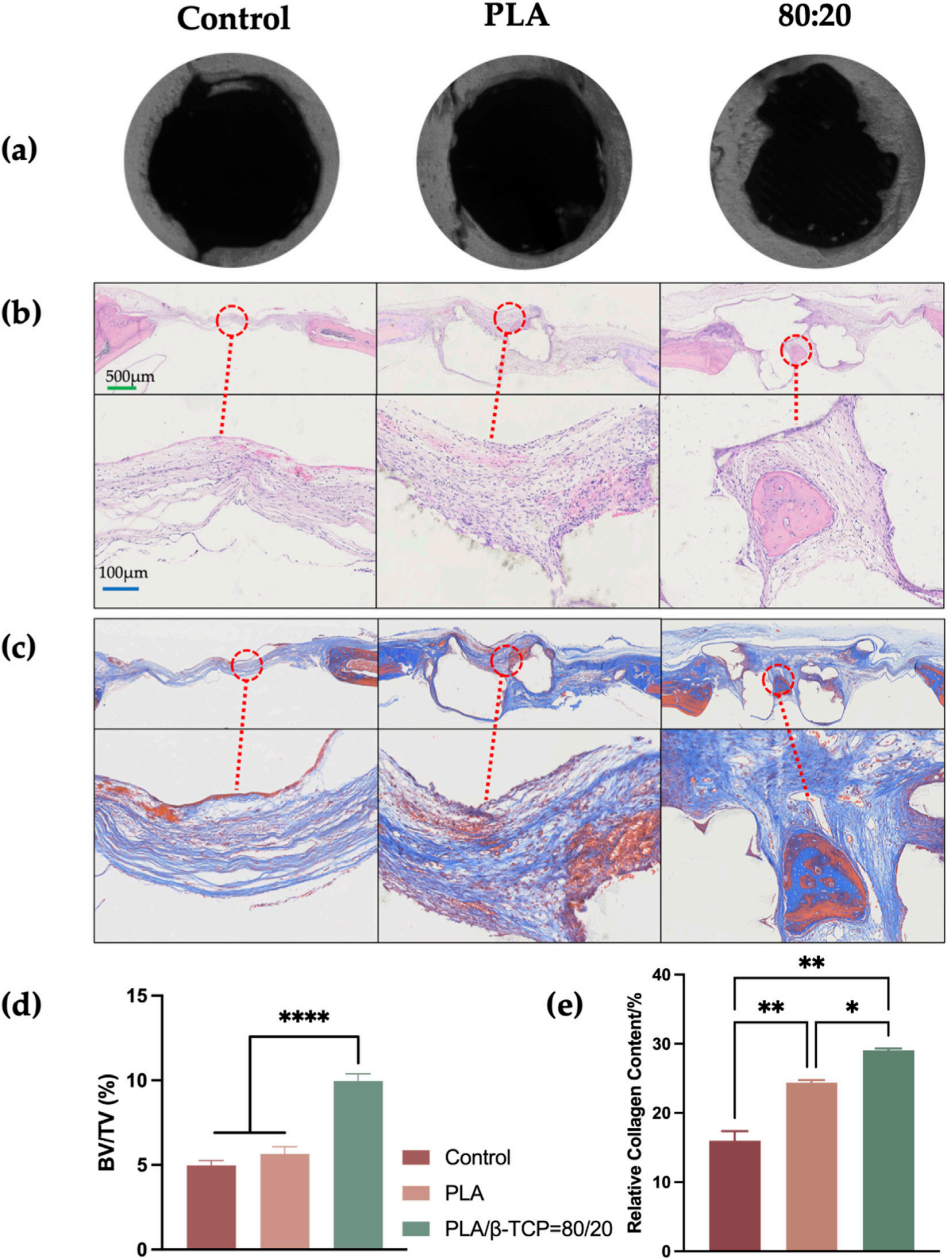
Micro-CT and histological evaluation of bone formation *in vivo*. **(a)** Representative micro-CT images of the control, PLA, and 80:20 groups. **(b)** H&E staining of decalcified sections. **(c)** Masson’s trichrome staining of decalcified sections. **(d)** Quantitative analysis of micro-CT results. **(e)** Quantitative analysis of collagen formation. (green bar = 500 μm, blue bar = 100 μm).

At 4 weeks post-implantation, micro-CT analysis (Figures 14a,d) revealed the most prominent bone regeneration in the 80:20 group, followed by the PLA group, with minimal new bone observed in the control group. In the 80:20 group, newly formed bone was clearly visible, primarily distributed along the periphery of the defect, whereas little to no bone formation was evident in the central region. This spatial pattern may be at-tributed to the centripetal migration of osteogenic cells from the defect margins. Even with scaffold support, coverage of the central area within 4 weeks may remain limited. In the control group, lacking structural support, cell migration appeared further impeded, resulting in minimal bone formation even at the periphery.

H&E staining (Figure 14b) demonstrated dense inflammatory cell infiltration at the defect margins in all groups, with limited trabecular bone formation and no organized bone architecture. In both the PLA and 80:20 groups, scaffold remnants were present and surrounded by fibrous connective tissue, showing good integration with the host bone. Notably, in the 80:20 group, newly formed trabeculae were observed in the central scaffold region, while no significant bone formation was detected within the central area of PLA scaffolds.

Masson’s trichrome staining (Figure 14c) showed a larger area of collagen deposition (blue staining) in the 80:20 group compared to the PLA group. Collagen fibers and new bone matrix were evident both centrally and peripherally within the 80:20 scaffolds, whereas in the PLA group, collagen deposition was minimal and more red-stained regions (indicative of muscle or inflammatory tissue) were observed. Quantitative analysis of Masson-stained sections (Figure 14e)confirmed superior bone regeneration in the 80:20 group, with statistically significant differences compared to both the PLA and control groups.

## 4 Discussion

Highly porous scaffold materials serve as the foundational framework in BTE, providing structural support for cell adhesion and mineralized matrix deposition. Extensive research has been dedicated to optimizing these scaffolds to simultaneously achieve excellent biocompatibility, tailored mechanical and degradation properties, biodegradability, processability, as well as osteoconductive and osteoinductive capabilities (Koons et al., 2020). 3D printing offers a highly reproducible, computer-guided layer-by-layer additive manufacturing process, demonstrating great potential in fabricating scaffolds with complex architectures, tunable geometries, and controllable porous structures—features that are difficult or impossible to achieve using conventional techniques such as freeze-drying or gas foaming (El-Fiqi, 2023). PLA exhibits excellent formability but limited bioactivity, whereas β-TCP possesses osteoconductivity and degradability but insufficient mechanical strength. Therefore, combining the two can partially reconcile the requirements for mechanical support and osteoinduction (Park, 2015; Sohn and Oh, 2019). Although previous studies have reported the effects of varying PLA/β-TCP ratios on scaffold performance, comprehensive investigations integrating systematic *in vitro* and *in vivo* evaluations with production feasibility analyses remain scarce.

Notably, the optimal PLA/β-TCP ratio differs among printing techniques due to variations in temperature control, shear environment, and material flow behavior. The printability of FDM materials is primarily governed by their rheological behavior, requiring pronounced shear-thinning and rapid cooling (Arrigo and Frache, 2022). Higher filler loadings generally reduce processability (Xu et al., 2023); however, melt flowability and interlayer bonding can be retained when anisotropic inorganic fillers reached ∼15% (Arrigo and Frache, 2022), whereas low-temperature or solvent-based printing requires lower β-TCP loadings to prevent agglomeration and nozzle clogging (Turnbull et al., 2018). In photopolymerization systems, filler particles affect light penetration and curing behavior, limiting dispersion and achievable loadings (Tumbleston et al., 2015; Babu et al., 2015). Hence, the “optimal” PLA/β-TCP ratio is not universal but depends strongly on the processing route and rheological compatibility of the composite.

In this study, commercially available PLA and β-TCP were blended via RAM and fabricated by FDM printing. The effects of different composite ratios on processing accuracy, mechanical properties, thermal characteristics, and biological behavior were systematically assessed, with the aim of providing experimental evidence and parameter references for the development of low-cost, clinically applicable bone scaffolds amenable to large-scale production, while also exploring optimization strategies for commercial FDM printers toward additive manufacturing of bioceramic composites, which are not optimized for biologically relevant materials such as β-TCP (Gregor et al., 2017).

Dissolving PLA in organic solvents and subsequently ultrasonically dispersing β-TCP into the solution followed by solvent evaporation is a commonly used method in many related studies for preparing PLA/β-TCP composite systems (Wang et al., 2023). However, this approach inevitably involves the use of toxic organic solvents such as dichloromethane and acetone, and the residual solvents may pose cytotoxic risks (Elhattab et al., 2022). Additionally, as we found in our trial, during solvent evaporation, β-TCP particles may still sediment, leading to phase separation within the composite system. In contrast, RAM enables the dry coating of low-concentration smaller (guest) particles onto larger (host) particles, achieving an acceptable blend uniformity (BU) even at low guest particle loadings without any involvement of toxic organic solvents (Gupta et al., 2022). In this study, the β-TCP powder exhibited a much smaller particle size (d50 = 2.061 μm) compared to the PLA powder (d_50_ = 281.982 μm). Within the RAM system, β-TCP particles were uniformly dispersed throughout the PLA matrix, as further evidenced by cross-sectional SEM imaging, EDS elemental mapping of the composite filaments, and micro-CT reconstruction of the printed scaffolds. Notably, the differences in particle size and physical properties between the two components did not lead to detectable agglomeration or phase-separation-induced inhomogeneity.

PLA exhibits relatively stable flow behavior in its molten state, making it resistant to decomposition or bubble formation. As a result, it can maintain a consistent diameter and shape during filament production, which is crucial to scalable filament production. Additionally, its favorable viscoelasticity in the molten state reduces the risk of breakage during extrusion (Chen and Smith, 2021). The incorporation of fine β-TCP particles may lead to a reduction in the shear viscosity of the composite system. Elhattab et al. (Elhattab et al., 2022) found that, under identical temperature and frequency conditions, both the elastic modulus and viscous modulus of β-TCP/PLA were lower than those of pure PLA. At the same temperature, PLA exhibited a higher storage modulus (G′) compared to β-TCP-PLA, which can be attributed to the strong intermolecular interactions between PLA chains. However, the addition of β-TCP particles in the composite disrupts these interactions, exhibiting a “lubricating” effect under high shear viscosity. As the mass fraction of β-TCP increases, the flowability of the filament during extrusion also improves. Although during printing, materials with higher flowability can move more easily through the nozzle to the print bed, reducing the possibility of nozzle clogging (Polamaplly et al., 2019), increased flowability also makes it more difficult to maintain consistent filament diameter and shape during production. In our extrusion process, we observed that as β-TCP content increased, the extrusion flow rate also rose. Variations in extrusion and nozzle temperature were required to accommodate the distinct rheological behavior of different composite formulations. Pure PLA exhibited relatively high melt viscosity, necessitating a higher nozzle temperature to promote adequate melt flow and stable extrusion. In contrast, the incorporation of β-TCP particles reduced the overall viscosity and increased melt flowability, enabling extrusion at slightly lower nozzle temperatures to prevent over-extrusion, filament spreading, and loss of geometrical fidelity. Accordingly, fine temperature adjustments were applied to maintain extrusion stability and preserve interlayer bonding across compositions. The 70:30 group was still suboptimal for filament extrusion even after we have adjusted the extrusion temperature, it became necessary to modify the rotational speed of the filament feed rollers to ensure diameter uniformity. When the β-TCP content reached 30%, the flowability of the composite system was too excessive to maintain the shape of the molten amorphous state prior to rapid solidification and the consistency of filament diameter and shape. As a result, the filament’s geometry became increasingly difficult to control. Additionally, the agglomeration of β-TCP particles further hindered diameter control. And β-TCP contents of 30 wt% and above will result in increased filament brittleness, which makes it highly prone to breakage during spool winding and unsuitable for large-scale industrial filament extrusion. Considering these processing limitations, this study did not explore composite ratios beyond 30 wt% β-TCP.

Across studies, a trade-off exists between the mechanical and biological performance of PLA/β-TCP composites. Moderate inorganic particle contents act as nucleating agents promoting the crystallization of polymer composites, which increases stiffness, whereas higher contents lead to particle agglomeration, interlayer defects, and brittleness (Liang, 2013). Conversely, bioactivity often continues to improve up to 25 wt% β-TCP due to increased Ca^2+^/PO_4_^3-^ ion release and buffering of acidic degradation products (Ftiti et al., 2024). In this study, when the β-TCP content was ≤20%, both the crystallinity and compressive strength of the composite system increased with rising β-TCP content. Gay et al. (Gay et al., 2009) found that the incorporation of HA nanoparticles into a PLLA matrix significantly enhanced the compressive strength and stiffness of the PLLA composites. Abbas et al. (Abbas et al., 2021) reported that uniformly distributed nano/micro β-TCP particles act as a micro-framework that blunts crack propagation and dissipates interfacial energy. However, this strengthening effect is mainly evident at low filler contents. At higher loadings, particle–polymer interactions restrict macromolecular mobility, shifting the fracture mode from ductile to brittle (Liang, 2013). As confirmed by the freeze-fracture SEM and EDS analyses, the 70:30 group exhibited localized β-TCP agglomeration. Such agglomerated “hard clusters” are readily formed through van der Waals attraction and act as preferential sites for stress concentration, facilitating microcrack initiation and propagation. Once cracks nucleate at these ceramic-rich regions, the weakened interfacial bonding accelerates failure, ultimately resulting in premature brittle fracture. (Mohammadi Zerankeshi et al., 2022). Consequently, the compressive strength of the 70:30 composites was notably reduced, and extensive through-cracks were observed along the loading direction.

The hydrated specimens showed interesting compressive behaviors. For pure PLA, short-term water absorption slightly reduced compressive strength, which may be attributed to water-induced plasticization of the polymer chains, lowering stiffness and facilitating local deformation (Chandran et al., 2021). However, β-TCP-containing samples exhibited a slight increase in compressive strength after hydration. Similar hydration-induced strengthening effects have been reported in multiphase composite systems such as concrete, where pore water partially replaces compressible air and behaves as an incompressible phase with a higher elastic modulus (Candelaria et al., 2021; Wang et al., 2022). In addition, the intrinsically hydrophilic nature of β-TCP enables preferential water uptake at the particle–matrix interface, partially masking the slight compressive strength reduction associated with the hydration-induced plasticization of the hydrophobic PLA matrix. As a result, the net effect manifests as a modest enhancement in compressive strength after hydration, particularly at moderate ceramic contents.

(Martínez-Vázquez et al., 2014) observed in their study on the flexural behavior of β-TCP scaffolds infiltrated with PLA that both flexural strength and toughness were significantly enhanced after polymer infiltration. This improvement was primarily attributed to the extraordinary strengthening provided by the impregnation process, as well as a crack-bridging toughening mechanism generated by polymer fibrils. In the tensile tests conducted in this study, the toughening mechanism of PLA microfibrils enabled significant internal material elongation during stretching, with filamentous structures visibly present at the fracture ends. However, the incorporation of β-TCP particles disrupted this tensile reinforcement by weakening the intermolecular interactions between PLA chains, resulting in minimal elongation at the fracture surfaces. This effect was particularly pronounced in the 70:30 group, where the tensile fracture strain dropped sharply to only 1.07%. From the perspective of mechanical performance, the strong inhibition of cold crystallization (high Tcc, low ΔHcc) caused by high β-TCP content is one of the key factors leading to extreme embrittlement under tensile loading (Ftiti et al., 2024). This limits PLA’s ability to dissipate energy through stress-induced crystallization under load. The high crystallinity of the 70:30 group may explain the climb of its Young’s modulus compared with the 80:20 group, but at the same time results in the lowest tensile strength and elongation at break. In addition, the decrease in Tg observed from DSC suggests that the interfacial bonding may not be particularly strong (Kim et al., 2007). The drastic drop in tensile strength and elongation at break directly reflects that poor interfacial adhesion is a key factor limiting composite performance (Turcsányi et al., 1988)—especially when β-TCP content reaches 30%, where SEM images of tensile fracture surfaces reveal interfacial debonding between the inorganic filler and the PLA matrix. The reinforcing effect of β-TCP (in compression) partially offsets the interfacial issue at low to medium contents (wt%≤20%). Under tension, however, it is completely masked by the interfacial problem.

β-TCP exhibits excellent hydrophilicity (Hu et al., 2014). As the proportion of β-TCP in the PLA matrix increases, the surface roughness of the samples increases and the water contact angle decreases. This structural alteration has a direct impact on cell affinity, as reflected in cellular adhesion behavior (Davison et al., 2014). Compared to the smooth surface of pure PLA, the rougher surface of PLA/β-TCP scaffolds presents a higher specific surface area and a more complex three-dimensional microenvironment, which better supports the extension of filopodia-like structures from rBMSCs. Another underlying mechanism may involve the influence of hydrophilicity on protein adsorption behavior. Enhanced surface wettability may increase the amount of protein adsorbed and promote a favorable protein conformation, thereby facilitating cell adhesion (Chen S. et al., 2018).

In this study, results from live/dead cell staining and cytoskeleton staining demonstrated that all scaffold groups exhibited good biocompatibility. After 7 days of osteogenic induction, ALP staining revealed significantly higher ALP expression in rBMSCs co-cultured with β-TCP-containing scaffolds compared to the pure PLA group, with staining intensity showing an increasing trend alongside rising β-TCP content.

RUNX2 is widely recognized as an early osteogenic differentiation marker and is frequently assessed *in vitro* by qPCR or Western blot during osteoinduction experiments (López-Serrano et al., 2024). As a key transcription factor regulating the differentiation of mesenchymal stem cells (MSCs) into the osteoblastic lineage, RUNX2 promotes osteogenesis by inducing the expression of collagen-related genes such as COL1A1, COL1A2, and SPP1 (Cui et al., 2025). In this study, rBMSCs co-cultured with the composite scaffolds for 7 days showed increased protein expression of Runx2 and elevated mRNA levels of both COL1A1 and Runx2, especially in the 80:20 and 70:30 groups. Osterix (Osx), a downstream transcription factor and a mid-stage osteogenic marker (Chan et al., 2021), showed no significant intergroup difference after 7 days of induction. Although ARS at day 14 did not reveal significant statistical differences between groups, results at day 28 mirrored the trends observed in ALP staining. This indicates that the incorporation of β-TCP not only enhances the expression of early osteogenic markers such as ALP but also promotes late-stage mineralization capacity.

In the *in vivo* experiments, evaluations conducted at postoperative week 4 using Micro-CT, H&E staining, and Masson staining demonstrated that the composite scaffold group (80:20) exhibited superior performance in bone defect repair compared to the pure PLA and blank control groups. Micro-CT analysis revealed a significant accumulation of newly formed bone tissue at the periphery of the defect site in the 80:20 group, while bone formation was still absent in the central region. This suggests that even with material support, osteoblast migration and bone formation in tissue-engineered scaffolds predominantly occur at the margins. This observation is consistent with previous findings on osteoblast migration patterns, which indicate that osteoblasts typically migrate from the periphery to the center, and bone formation in the central region generally requires more time (O’Brien, 2011).

H&E staining further confirmed the tissue regeneration status. Although some degree of osteogenic response was observed at the defect edge in all groups, only the 80:20 group showed evident formation of new trabeculae in the central region of the scaffold. This indicates that the composite material more effectively supports deep bone regeneration. In contrast, the PLA group exhibited a lack of osteogenic signs in the central zone, suggesting that PLA alone may be insufficient to provide the necessary support or inductive microenvironment for osteogenesis. Masson staining results aligned with the H&E staining trends, illustrating collagen fiber deposition. As a key early indicator of bone formation, the 80:20 group exhibited a larger area of blue-stained collagen fibers, including new collagen formation in the scaffold’s central region, reflecting enhanced bone matrix synthesis activity.

From a clinical perspective, large-scale translation of PLA/β-TCP scaffolds remains limited by regulatory approval, manufacturing reproducibility, quality control, and cost-effectiveness (Zhao et al., 2024). This study offers guidance for addressing these challenges by optimizing extrusion and nozzle temperature parameters to standardize filament fabrication and ensure consistent scaffold performance. The established process–property relationships also provide a reference for optimizing FDM bioprinters tailored to biomedical composites. Future work integrating real-time monitoring and automated temperature calibration may further enhance production reliability, bridging the gap between laboratory research and scalable clinical manufacturing of 3D-printed bone scaffolds.

## 5 Conclusion

This study successfully developed a novel β-TCP/PLA composite scaffold utilizing FDM technology. A non-toxic RAM method was employed to uniformly blend the raw materials, yielding composite filaments with excellent processability. Our findings indicate that the 80:20 β-TCP/PLA ratio exhibited optimal performance across various aspects, including forming quality, mechanical properties, biocompatibility, osteoinductive capability, and *in vivo* bone repair efficacy.

The incorporation of β-TCP significantly enhanced the scaffold’s surface roughness and hydrophilicity, which in turn promoted improved cell adhesion and augmented the expression of osteogenesis-related proteins and genes. *In vivo* experiments further corroborated the superiority of the 80:20 group in bone defect healing. This composite scaffold demonstrates considerable potential for structural design and promising application prospects, offering a viable pathway for the development of personalized and load-bearing bone repair materials.

## Data Availability

The raw data supporting the conclusions of this article will be made available by the corresponding author upon reasonable request.
